# Therapeutic Outcomes of Isatin and Its Derivatives against Multiple Diseases: Recent Developments in Drug Discovery

**DOI:** 10.3390/ph15030272

**Published:** 2022-02-22

**Authors:** Rameshwar S. Cheke, Vaishali M. Patil, Sandip D. Firke, Jaya P. Ambhore, Iqrar A. Ansari, Harun M. Patel, Sachin D. Shinde, Visweswara Rao Pasupuleti, Md Imtaiyaz Hassan, Mohd Adnan, Adel Kadri, Mejdi Snoussi

**Affiliations:** 1 Department of Pharmaceutical Chemistry, Dr. Rajendra Gode College of Pharmacy, Malkapur 443101, Maharashtra, India; ambhorejp02@gmail.com; 2 Department of Pharmaceutical Chemistry, KIET School of Pharmacy, KIET Group of Institutions, Delhi-NCR, Ghaziabad 201206, Uttar Pradesh, India; vaishuwise@gmail.com; 3 Department of Pharmaceutical Chemistry, R. C. Patel Institute of Pharmaceutical Education and Research, Shirpur 425405, Maharashtra, India; sandipfirke@rediffmail.com (S.D.F.); ansariiqrar50@gmail.com (I.A.A.); hpatel_38@yahoo.com (H.M.P.); 4 Department of Pharmacology, Shri. R. D. Bhakt College of Pharmacy, Jalna 431213, Maharashtra, India; sdshinde8390@gmail.com; 5 Department of Biomedical Sciences and Therapeutics, Faculty of Medicine & Health Sciences, University Malaysia Sabah, Kota Kinabalu 44800, Sabah, Malaysia; 6 Department of Biochemistry, Faculty of Medicine and Health Sciences, Abdurrab University, Pekanbaru 28291, Riau, Indonesia; 7 Centre for International Collaboration and Research, Reva University, Rukmini Knowledge Park, Kattigenahalli, Yelahanka, Bangalore 560064, Karnataka, India; 8 Centre for Interdisciplinary Research in Basic Sciences, Jamia Millia Islamia, Jamia Nagar, New Delhi 110025, India; mihassan@jmi.ac.in; 9 Department of Biology, College of Science, University of Hail, Hail P.O. Box 2440, Ha′il 2440, Saudi Arabia; drmohdadnan@gmail.com (M.A.); snmejdi@yahoo.fr (M.S.); 10 Faculty of Science of Sfax, Department of Chemistry, University of Sfax, B.P. 1171, Sfax 3000, Tunisia; lukadel@yahoo.fr; 11 Faculty of Science and Arts in Baljurashi, Albaha University, P.O. Box 1988, Albaha 65527, Saudi Arabia; 12 Laboratory of Genetics, Biodiversity and Valorization of Bio-Resources (LR11ES41), University of Monastir, Higher Institute of Biotechnology of Monastir, Avenue Tahar Haddad, BP74, Monastir 5000, Tunisia

**Keywords:** chemotherapeutic agent, anticancer drugs, isatin derivatives, drug design and development, heterocyclic compounds, therapeutic targeting

## Abstract

Isatin (1*H* indole 2, 3-dione) is a heterocyclic, endogenous lead molecule recognized in humans and different plants. The isatin nucleus and its derivatives are owed the attention of researchers due to their diverse pharmacological activities such as anticancer, anti-TB, antifungal, antimicrobial, antioxidant, anti-inflammatory, anticonvulsant, anti-HIV, and so on. Many research chemists take advantage of the gentle structure of isatins, such as NH at position 1 and carbonyl functions at positions 2 and 3, for designing biologically active analogues via different approaches. Literature surveys based on reported preclinical, clinical, and patented details confirm the multitarget profile of isatin analogues and thus their importance in the field of medicinal chemistry as a potent chemotherapeutic agent. This review represents the recent development of isatin analogues possessing potential pharmacological action in the years 2016–2020. The structure–activity relationship is also discussed to provide a pharmacophoric pattern that may contribute in the future to the design and synthesis of potent and less toxic therapeutics.

## 1. Introduction

**Figure 1:**
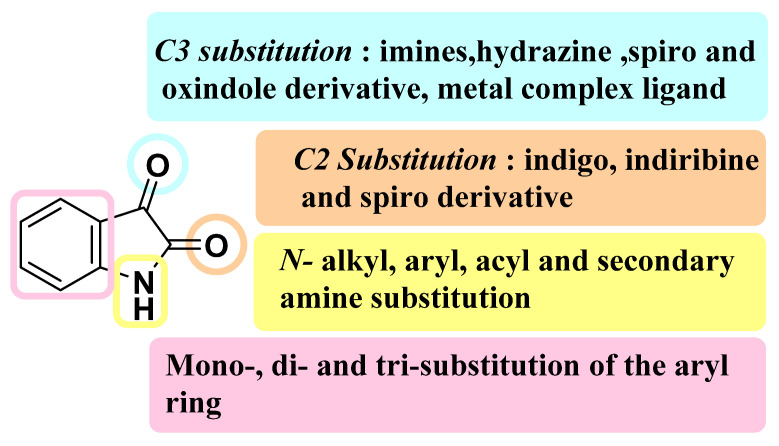
Probable substitution possible on isatin nucleus.

Heterocyclic entities are denoted as a vital class of organic compounds with wide biological and pharmacological potency [1,2]. In view of wide-ranging biological activities, these heterocyclic analogues, either individually or as fused forms, are consistently utilized by scientists and research groups for the design of the novel candidates [3]. Among them, isatin, or (1*H*-indole-2,3-dione), has a wide spectrum of pharmacological potential and is thus appraised as a fortunate bioactive heterocyclic moiety. The isatin molecule is comprised of six- and five-membered cyclic planar rings; in contrast, the six-member ring has an aromatic character while the five-member ring possesses antiaromatic properties. Isatin was first brought to light by L. Erdman and A. Laurent in 1841 from the oxidation of indigo dye by nitric and chromic acids furnished as orange monoclinic crystals [4]. This isatin was considered a synthetic moiety for almost 140 years until it was isolated from the plants of *Isatis genus* [5]*, Calanthe discolor* LINDL [6], the fruit of the cannon ball tree *Couroupita guianensis* Aubl [7], as a constituent of the secretion from the parotid gland of Bufo frogs [8], and as metabolic derivatives of adrenalin in humans [9]. Moreover, isatin was found to be a component of coal tar while its derivatives fall out in variable dye, pharmaceuticals, and agriculture chemicals [7]. Nowadays, isatin has achieved a position in the design and development of medicinally active analogues because of its fortunate electronic nature. The possible substitutions for isatin hybrids are depicted in Figure 1.

**Figure 2:**
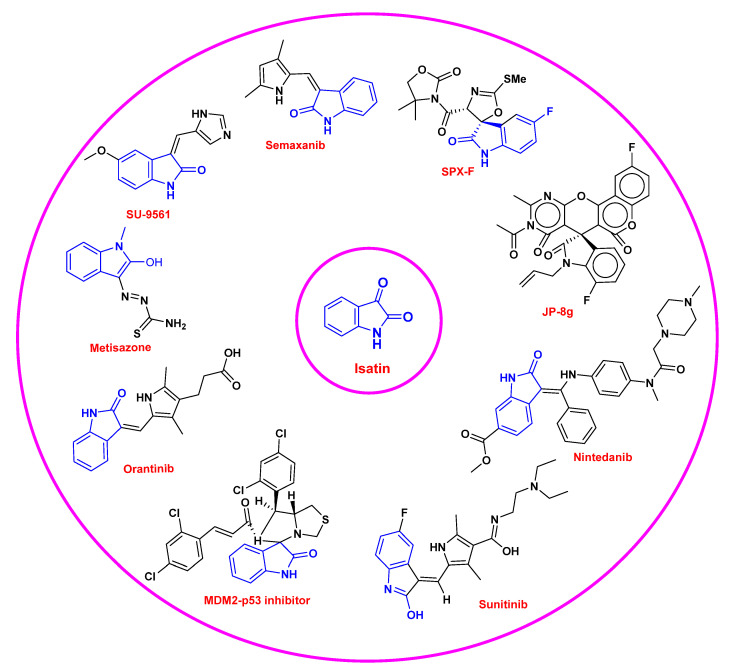
Marketed candidates containing isatin moiety.

Isatin derivatives revealed a fascinating array of pharmacological activities, such as anticancer [10], anti-HIV [11], antiviral [12], antitumor [13], antifungal [14], antimalarial [15], antioxidant [16], anti-inflammatory [17], antimicrobial [18], analgesic [19], anticonvulsants [20], and so on. Several conventional analogues of isatin are currently being used for medicinal purposes, and some representative examples of marketed drugs containing isatin scaffolds against multiple diseases/disorders are displayed in Figure 2. In the last 15–20 years, owing to the therapeutic efficacy of thee isatin moiety, a vast number of synthetic approaches have been developed to synthesize isatin derivatives for the evaluation of their biological potential. Recently, several medicinal chemists undertook the privilege of reviewing the medicinal features of isatin, keeping in mind to provide a systematic understanding of isatin derivatives in relation to their pharmacological profile, either as an individual nucleus [21,22,23] or in combination [24] for the readers. Additionally, our research group has revisited the survey to unfold the biological potential of isatin and its derivatives [25].

The structure—activity relationship (SAR) connects the lead molecule and its biological action and is useful for medicinal chemists in designing strategies to insert different moieties as bioactive analogues to further explore their pharmacological action. In recent years, chemists have been working hard towards the optimization of synthesis methods and properties of newly obtained isatin analogues that may be used to explore different pharmacological actions, for instance, improving potency, reducing toxicity, and ensuring sufficient bioavailability. In view of this, we plan to revisit and report the synthesis of isatin derivatives for their different pharmacological activities supported by molecular docking studies, a systematic exploration of the SAR trends using the reported literature by eminent researchers in the period 2016–2020 (recent 5 years).

## 2. Development of Isatin Derivatives as Promising Therapeutic Agents

### 2.1. Anticancer Activity of Isatin

**Figure 3:**
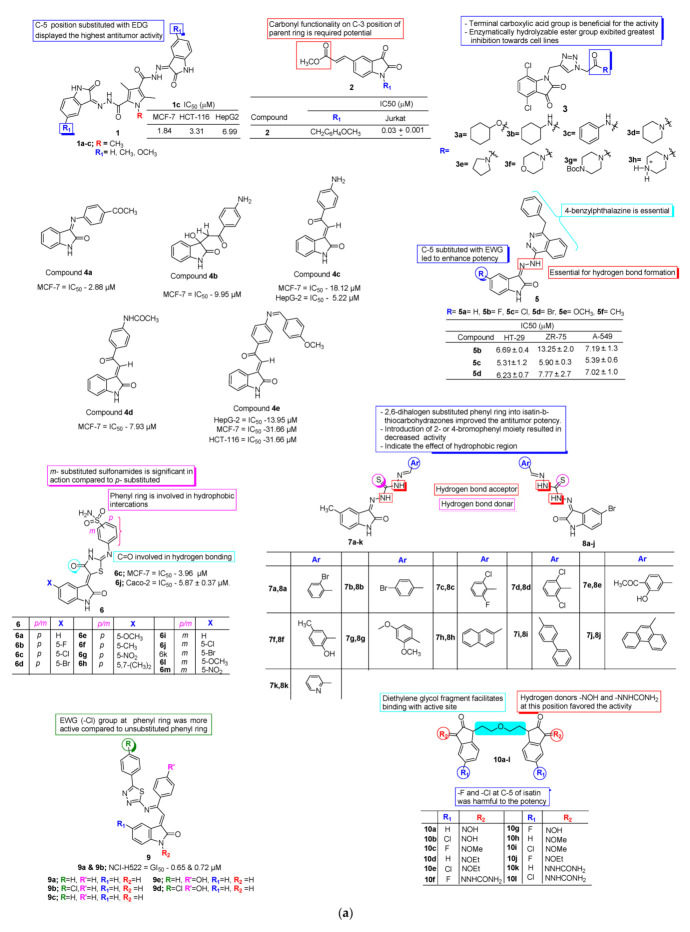

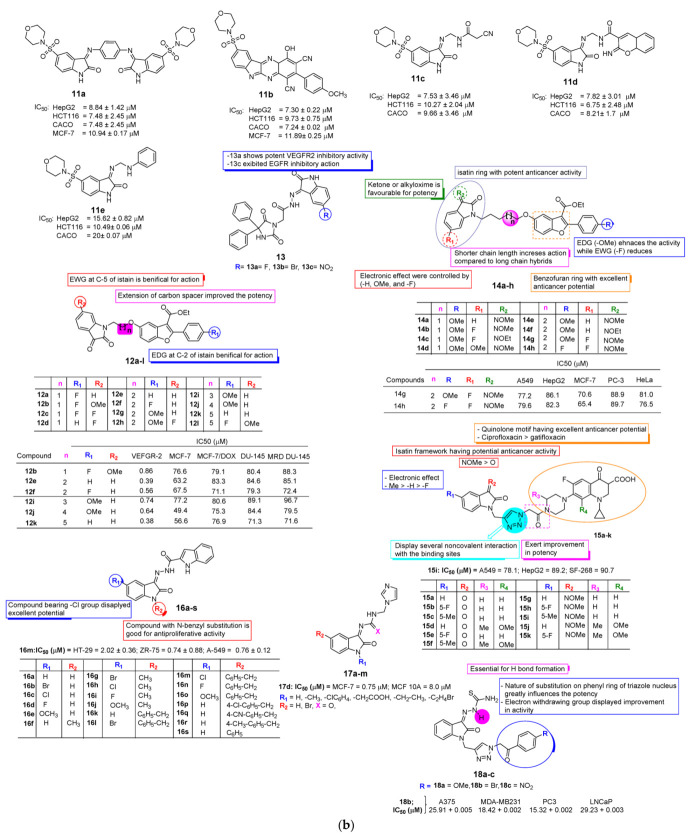
(**a**) SAR features of isatin derivatives exhibiting anticancer action. (**b**) Isatin derivatives exhibiting anticancer action and their SAR features.

To develop potent anticancer candidates, Ibrahim et al. [26] in 2016 reported four series of bis-isatins with hydrazide linkers along with their anti-proliferative activity by evaluating their cytotoxic potential against HepG2 (liver), MCF-7 (breast), and HCT-116 (colon) cancer cell lines. The research group emphasized that most of the analogues from the series exhibited moderate to weak potential against tested human tumor cell lines. In contrast, compound **1** displayed potent antiproliferative activity against MCF-7 and HCT-116 with IC_50_ values of 1.84 µM and 3.31 µM, respectively, compared to the standard drug doxorubicin with IC_50_ values of 2.57 µM and 3.70 µM against cell lines MCF-7 and HCT-116, respectively. Moreover, compound **1** was also found to be significantly active against HepG2 with IC_50_ 6.99 µM, which is closer to standard doxorubicin at 3.56 µM. The results highlighted that compound **1** with two 5-methoxy isatin rings linked via a 1,3,5-trimethyl-1Hpyrrole-2,4-dicarbohydrazide linker found excellent antitumor potential against tested human tumor cell lines compared to the standard. A cell-cycle analysis study of analogue **1** with the most sensitive cell line, MCF-7, displayed noteworthy G2/M arrest coupled with an elevation in the percentage of cells in the pre-G phase, which is indicatory of apoptosis (Figure 3a).

Yu-Ou Teng et al. [27] in 2016 examined and synthesized forty-three new di/trisubstituted isatin analogues against human T lymphocyte cells Jurkat via in vitro MTT assay with camptothecin as the positive control. Among them, compound **2** displayed potent cytotoxic action with an IC_50_ value of 0.03 µM, which is 330-fold greater compared to the parent isatin framework. Further investigations such as changes in cell morphology and annexin V/PI staining confirmed that analogue **2** inhibited the proliferation of Jurkat cells by inducing apoptosis through the mitochondrial apoptotic pathway. Moreover, active compound **2** employs their inhibition effect on many cancer cells. SAR analysis revealed that the substitution of 1-benzyl and 5-[trans-2-(methoxycarbonyl) ethen-1-yl] greatly improved their cytotoxic potency. The obtained results suggested that compound 2 has a broad range of anticancer effects against human Jurkat cells (Figure 3a).

Bin Yu et al. [28] in 2016 designed a series of novel isatin/triazole conjugates. From the preliminary investigation of the biological potential of isatin-triazole analogues, they suggested that most of the analogues of conjugates found moderate to good growth inhibition towards MGC-803 cells and remained less lethal to normal cells HL-7702 and GES-1. Among these, compounds **3** exhibited excellent potential towards MGC-803 cells, with an IC_50_ value 9.78 of µM, was less toxic towards the normal cells HL-7702 and GES-1 with IC_50_ values of 40.27 µM and 35.97 µM, respectively, and induced apoptosis via multiple mechanisms, in addition to the migration of MGC-803 Cells (Figure 3a).

In 2017, Ammar et al. [29] synthesized isatin-linked chalcone analogues (Figure 3a) with an aim to investigate the cyclin-dependent kinase 2 (CDK2) inhibitory properties and cytotoxic potency against human carcinoma cell lines HepG-2, MCF-7, HCT-116, and the MCF-12A breast cell line. All designed hybrids were found to be active against tested cell lines with IC_50_ values ranging from 2.88 to 62.88 µM as compared to the positive control imatinib. In contrast, compounds **4a**–**d** displayed excellent potential with IC_50_ 2.88 to 18.12 µM against three cell lines when compared to the standard, whereas compound **4e** exhibited moderate potential against HepG-2, MCF-7, and HCT-116 with IC_50_ 13.95, 31.66, and 11.78 µM, respectively. Compound **4a** with the acetyl group displayed the highest potential against MCF-7 followed by compound **4d** with the acetamide group, while compound **4c** was most effective against the HepG-2 cell line followed by **4d**, **4a**, and **4b**. For a better understanding, a docking study was also performed against CDK2 (PDB ID: 4BCQ), which revealed that compound **4c** shows the best energy score and binding interactions with amino acid residues (Phe A:80 and Leu A:83) in the active site of CDK2/CyA, which demonstrates it to be a potential candidate against cyclin-dependent kinase 2, i.e., it may be involved in anticancer activity [29].

Novel isatin-benzoazine molecular hybrids (**5a**–**f**, Figure 3a) were synthesized in 2017 by Abdel-Aziz et al. [30] The furnished analogues were screened for their antiproliferative activity towards HT-29 (colon), ZR-75 (breast) in A-549 (lung) human cancer cell lines. Among the series, isatin-clubbed phthalazine analogues **5b**–**d** emerged as promising antiproliferative candidates. In contrast, compound **5c** induced apoptosis by enhancing the caspase 3/7 activity (about 5-fold at 15 µM conc.) against the A-549 human cancer cell line. Furthermore, compound **5c** has shown an increase in the G1 phase and a decrease in the S phase as well as G2/M phases in the cell cycle assay and shows an IC_50_ value of 9.5 µM against NCI-H69AR cancer cell lines.

In 2017, Eldehna et al. [31] reported novel 4/3-((4-oxo-5-(2-oxoindolin-3-ylidene) thiazolidin-2-ylidene) amino) benzenesulfonamides for their carbonic anhydrase inhibitory and anticancer potential. All synthesized analogues were screened for growth inhibition against MCF-7 (breast) and Caco-2 (colorectal) cancer cell lines. Among the series, compound **6c** has shown excellent growth inhibition against the MCF-7 cell line with IC_50_ 3.96 ± 0.21 µM, while compound **6j** was found most active against Caco-2 cells with IC_50_ 5.87 ± 0.37 µM. Compound **6c** induces apoptosis in MCF-7 via enhancing proapoptotic protein Bax and reducing the antiapoptotic protein Bcl-2 as well as increasing the regulation of caspase-9/3 levels (Figure 3a).

A new series of isatin-b-thiocarbohydrazones were synthesized in 2017 by Gabr et al. [32] via the microwave-assisted technique, and were assessed for their antitumor activity against Hela (cervical cancer) and COS-7 (kidney fibroblast cancer) cell lines. From the series, compounds **7c**–**d**, **8c**, and **8e** displayed excellent potency against Hela cell lines while analogues **7c**–**d**, **7k**, **8c**, and **8e** exhibited noteworthy potential against COS-7 cell lines. In contrast, compound **7d** increases the lifespan of mice injected with EAC to a higher extent. SAR analysis revealed that compounds **7c**–**d, 8c**, and **8e** with phenyl ring 2,6-dihalogen substituted on the isatin-b-thiocarbohydrazones complex enhances the antitumor potential against Hela and COS-7 cell lines. Moreover, compounds **7c** and **8c,** with a 2-chloro-6-fluorophenyl moiety, displayed greater potential as compared to the analogues with the 6-dichlorophenyl counterpart. In addition, the incorporation of 3-hydroxy-4- methoxyphenyl substitution into the isatin-b-thiocarbohydrazones **7e** and **8e** exhibited promising results against both cell lines, while the substitution of the pyridin-2-yl moiety to the 5-methylisatin-b-thiocarbohydrazone **7d** led to increased potency against both Hela and COS-7 cell lines (Figure 3a).

Kiran Gangarapu et al. [33] designed and synthesized a series of novel 3-(2- (*p*-substituted)-2-((5-phenyl-1,3,4-thiadiazol-2-yl) imino)-2- (*p*-substituted) ethylidene) indolin-2-one and 5-substituted-5′-substituted phenyl-2′,4′-dihydrospiro[indoline3,3′-pyrazol]-2-one derivatives (**9a**–**d**, Figure 3a). The synthesized analogues were screened for anticancer activity against a panel of 60 cancer cell lines. The obtained results show 3-(2- Phenyl-2-((5-phenyl-1,3,4-thiadiazol-2-yl) imino) ethylidene) indolin-2-one (**9a**) and 3-(2-((5-(4-chlorophenyl)-1,3,4-thiadiazol-2-yl) imino)-2-phenylethylidene) indolin-2-one (**9b**) could exhibit potent antitumor activity against non-small cell lung cancer (NCI-H522) with GI_50_ (MG-MID) values of 0.65 and 0.72 µM, respectively.

In 2018, Li and co-workers [34] reported the antitumor, anti-mycobacterial, and anti-HIV potential of some novel diethylene glycol tethered bis-isatin analogues (**10a**–**l**, Figure 3a). The molecular hybrids were assessed for their anticancer potency against eight human cancer cell lines, namely, HepG2, Hela, HCT-116, A549, DU145, SKOV3, MCF-7, and MCF-7/DOX (Doxorubicin-resistant MCF-7) via SRB assay using etoposide as the positive control. It was suggested by the research group that eight bis-isatin analogues exhibited excellent potency against all screened human cancer cell lines with IC_50_ values between 8.32 and 49.73 μM. In contrast, analogue **10a** displayed broad-spectrum activity >2.5-fold higher than the standard drug, etoposide, against Hela, HCT-116, A549, and drug-resistance MCF-7/DOX cell lines. Compound **10a** was further subjected to tubulin inhibitory assay, suggesting that bis-isatin hybrids may display anticancer potential via the inhibition of tubulin. SAR analysis displayed that bis-isatin analogues substituted at C-3 and C-5 positions of isatins could exhibit potential anticancer activity against tested human cancer cell lines.

Another study accomplished by Ammar et al. [35] in 2018 developed a series of novel 5- (morpholino sulfonyl) isatin derivatives **(11a**–**e**, Figure 3b) with potential EGFR inhibitory activity. The developed analogues were evaluated for their cytotoxic effects against HepG2, HCT116, CACO, and MCF-7 cancer cell lines using SRB assay and doxorubicin as a standard drug. The bis-derivative, analogue **11a,** exhibited good anticancer potential, while compound **11b** with substitution of the hydroxy group at position 1 and dicarbonitrile at position 2 and 4 of quinoxaline was found to possess significant cytotoxic activity. Moreover, compounds **11c** and **11d,** on substitutions with the cyanoacetohydrazide and chromene-3-carbohydrazide framework, led to enhanced activity and was found to be active nearly to the reference analog. Compound **11e,** with substitution of the phenyl ring at position 3, exhibited greater potency, especially against HCT116 with an IC_50_ value of ≤10 µM. Most active analogues from the series were further observed to possess decent inhibitory potential against the EGFR tyrosine kinase. In contrast, compounds **11e** (IC_50_ 46 nM) and **11c** (IC_50_ 23 nM) in a cell-cycle analysis displayed cell accumulation at the G2/M phase and induced pri-G1 apoptosis. A docking study for the active analogues **11e** and **11c** has shown hydrogen bonding interactions with the amino acid residue Met769 in the active site of the EGFR-tyrosine kinase like the standard erlotinib [35].

In 2019, Yulin Zou [36] synthesized and estimated the VEGFR-2 inhibitory activity and in vitro anticancer potential of benzofuran-isatin conjugates **(12a**–**l**, Figure 3b) against MCF-7, MCF-7/DOX, DU-145, and MDR DU-145 cancer cell lines by MTT assay. The presented results suggested that seven analogues, **12b**, **12e**, **12f**, and **12i**–**k,** could display better potency with IC_50_ values of 47.6–96.7 μM against all tested cell lines compared to the positive control sunitinib. SAR results illustrate the carbon spacer between isatin and the benzofuran ring, the isatin motif substituted at C-3 and C-5 positions, and the incorporation of the *para*-substituted phenyl ring at the C-2 position of the benzofuran moiety as required features for significant VEGFR-2 inhibitory and anticancer potential. The higher VEGFR-2 and anticancer potential exhibited by the analogues had ketone species.

A series of fifteen novel derivatives of 5,5-diphenylhydantoin bearing benzylidene or isatin (**13a**–**c**) were designed and synthesized by Alkahtani et al. [37]. The synthesized analogues were evaluated for their anticancer, apoptosis-inducing activities, and EGFR and VEGFR-2 inhibitory potential. Compound **13a** exhibited potent VEGFR2 inhibitory activity with IC_50_ values of 0.09 mM, while compound **13c** has potential against EGFR with IC_50_ values of 0.37 mM. The hybrids **13a**–**c** exhibited broad-spectrum cytotoxic potential against the tested HeLa cell line with IC_50_ values of 18.5, 29, and 30 mM, respectively, compared to the positive control docetaxel with IC_50_ 100 mM. In addition, compound **13a** induced caspase-dependent apoptosis and reactive oxygen species (ROS) production at 5 and 10 mM. A molecular docking study also revealed that compounds **13a** and **13c** could show good hydrogen bonding interactions in the active sites of VEGFR2 and EGFR, respectively [37].

Xu et al. [38], in 2019, described the anticancer activity of benzofuran–isatin hybrids tied via pentylene and hexylene (**14a**–**h**). Furnished hybrids were screened for their anticancer potency against A549, HepG2, MCF-7, PC-3, and HeLa human cancer cell lines using a CCK-8 Assay Kit. The majority of the analogues were found active against tested cell lines, amidst compounds **14g** and **14h** with IC_50_ values of 77.2–88.9 μM and 65.4–89.7 μM, respectively. These compounds possessed broad-spectrum anticancer activity compared with the positive control vorinostat with IC_50_ values of 64.2 – >100 μM against all screened human cell lines. SAR analysis revealed that substitution at the C-3 and C-5 positions of the isatin moiety and benzofuran motif greatly affects the anticancer potency. The incorporation of the electronegative atom -F at the benzofuran motif and C-5 position of isatin could improve the potency while the electron-withdrawing group reduced the anticancer activity. Shorter pentylene-tethered hybrids exhibited greater potency as compared to the corresponding extended hexylene-linked hybrids (Figure 3b).

In 2019, Zhang et al. [39] analyzed the anticancer activity of eleven novel ciprofloxacin/gatifloxacin-1,2,3-triazole-isatin analogues (Figure 3b). The detection of anticancer potency was conducted on A549, HepG2, MCF-7, PANC-1, and SF-268 human cancer cell lines by a CCK-8 Assay Kit. It was suggested that the majority of the designed hybrids exhibited potency against A549, HepG2, and SF-268 cancer cell lines. In contrast, compound **15i** displayed broad-spectrum anticancer potential with IC_50_ values of 78.1–90.7 μM against A549, HepG2, and SF-268 cancer cell lines compared to the reference vorinostat with IC_50_ 71.1- > 100 μM. Results of in vitro and SAR studies revealed the fluoroquinolone framework is closely associated with the activity. Methoxyimino substitution at the C-3 position of the isatin nucleus might improve the activity. C-5-substituted electron-donating methyl was valuable to improve the potential while electron-withdrawing fluoro was unfavorable.

Recently, the antiproliferative activity of new isatin-based conjugates was assessed by Al-Wabli et al. [40] in 2020, against HT-29, ZR-75, and A-549 cell lines. From the prepared conjugates, compound **16m** exhibited excellent in vitro antiproliferative potency against all tested human cancer cell lines with average IC_50_ values of 1.17 µM, which is nearly seven-fold more than that of the reference sunitinib (IC_50_ 8.11 µM). Further investigation suggested that compound **16m** increases the number of cells in the G1 phase, with a resulting reduction in those cells in the G2/M and S phases. Moreover, compound **16m** reduced the amount of the phosphorylated retinoblastoma protein significantly along with increased BAX expression and activated caspase-3, as displayed by the observed increase in cleaved caspase-3 levels. Form the obtained results, the SAR study showed the isatin moiety substituted with an N–benzyl ring seems to be fortunate for antiproliferative activity (Figure 3b). 

One-pot multicomponent synthesis of isatin-derived imidazole as dual-purpose hybrids against inflammation and cancer was demonstrated by Kumar et al. [41] in 2020. The anticancer activity was carried out on MCF-7 (breast cancer cell line) and MCF-10A (normal breast epithelial cells) by MTT assay. In contrast to the prepared hybrids, compound **17m** caused 40% cell death at 0.75 μM in MCF-7 cells, while 70% cell survival was noted at 8.0μM in MCF 10A cells. Compound **17m** exhibited excellent potential, which showed ~10-folds higher cytotoxicity against the breast cancer cell line (Figure 3b).

In 2020, Nazari and co-workers [42] reported the synthesis of triazol/spiroindolinequinazoline dione, triazol/indolin-3-thiosemicarbazone, and triazol/thiazol-indolin-2-one conjugates for their antitumor activity (Figure 3b). The furnished derivatives were assessed for their in vitro cytotoxic potential against cancer lines, namely, A375, PC3, LNCaP, MDA, MB231, and normal cell HDF. The majority of the compounds were found to be potent compared to the positive control. The triazol-linked oxindol-thiosemicarbazone analogue **18b** was found to be highly potent against all tested cancer cell lines with IC_50_ values between 15.32 and 29.23 μM compared to the reference etoposide. Further studies revealed that the compound **18b** is able to induce apoptosis by alteration of the Bax and Bcl2 balance. A SAR study suggested the importance of bromophenyl group substitution in the triazol/indolin-3-thiosemicarbazone nucleus for potent antitumor potential. The possible substitution pattern for potential anticancer activity is displayed in Table 1.

### 2.2. Anti-TB Activity of Isatin

**Figure 4:**
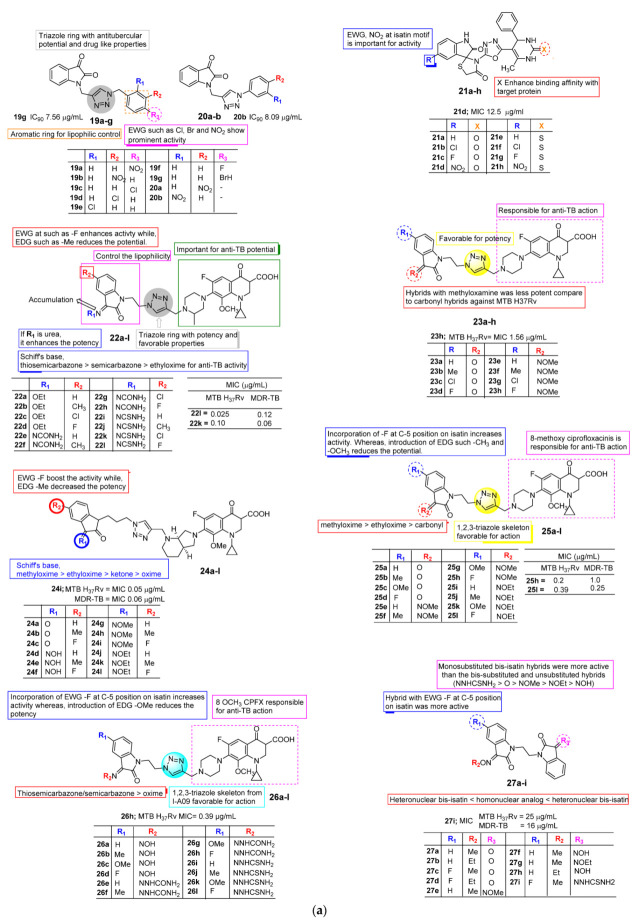

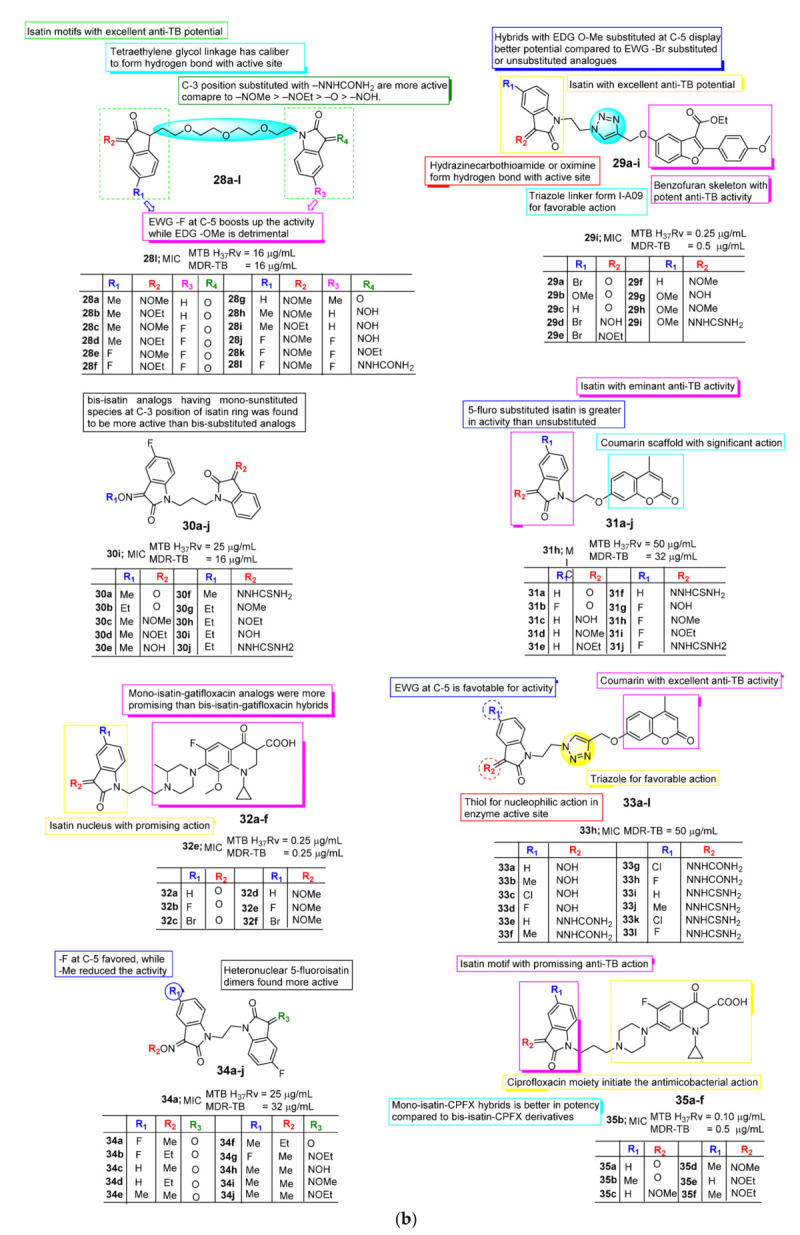

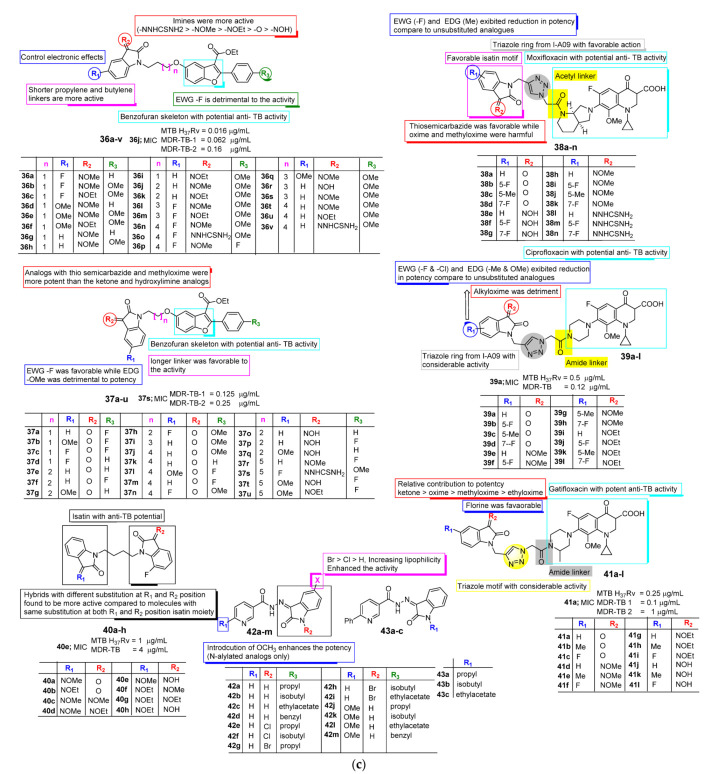
(**a**) Isatin analogues with potential anti-TB activity and identified SAR characteristics. (**b**) Isatin analogues with potential anti-TB activity and their SAR characteristics. (**c**) Isatin analogues with potential anti-TB activity and SAR characteristics.

In 2016, Shaikh et al. [43] reported the synthesis of some novel triazole-incorporated isatin derivatives (Figure 4a) for their antifungal, antitubercular, and antioxidant activity. The results showed that many synthesized hybrids have potent activity against MTB H37Ra. Among these compounds, **20b** exhibited the highest antitubercular potency with IC_90_ 7.56 μg/mL against MTB H37Ra compared to other synthesized analogues. Compound **19g** with bromo substitution at the *para* position of the phenyl ring was found to be the second active analogue with an IC_90_ value of 8.09 μg/mL. The anti-dormancy activity on dormant bacilli proposed that compounds **20b** and **19g** displayed higher anti-dormancy activity compared to other hybrids. A SAR study suggested that the *para*-substituted phenyl ring is important for antitubercular potential. When the *para* position of the phenyl ring is substituted by nitro and chloro species, the compounds display MIC values more than 30 μg/mL. Likewise, in the *para* position substituted by fluoro, it shows MIC 29.05 ± 0.09 μg/mL. Similarly, the substituted bromo group displayed excellent antitubercular potential with an MIC value of 8.09 ± 0.63 μg/mL.

Microwave-assisted synthesis of some novel spiro[indole-thiazolidines] hybrids for their antitubercular activity against H37RV were reported by Borad et al. [44] in 2016. Among the tested hybrids (**21a**–**h**, Figure 4a), compound **21d** exhibited better activity with MIC 12.5 mg/mL against the H37RV strain, which is near the MIC values of standard isoniazid compared to other prepared derivatives. 

Novel gatifloxacin-1H-1,2,3-triazole-isatin analogues (**22a**–**l**) were synthesized and assessed for their in vitro anti-mycobacterial potential against *M. tuberculosis* (MTB) H37Rv and MDR-TB by Xu et al. [45] in 2017. The research group emphasized that all tested analogues displayed excellent inhibitory potential against MTB H37Rv and MDR-TB with an MIC range of 0.025–3.12 mg/mL. In contrast, the thiosemicarbazone derivative **22l** proved to be the most active with MIC 0.025 mg/mL and 2–32 times more potent than that of the standards isoniazid (INH), gatifloxacin (GTFX), and rifamycin (RIF) with MIC values of 0.05, 0.78, and 0.39, mg/mL respectively, against MTB H37Rv. Meanwhile, compound **22k** exhibited excellent inhibitory potential with MIC 0.06 mg/mL against MDR-TB. A SAR study revealed the structural features of Schiff’s bases required for anti-TB potential as thiosemicarbazone > semicarbazone > ethyloxime. Substitution of electron-withdrawing species such as -F at the C-5 position of the isatin moiety enhances the anti-mycobacterial potential, whereas electron-donating species (-Me) reduce the general potency.

In 2017, Hu et al. [46] studied the antimycobacterial profile of eight 1H-1,2,3-triazole-tethered ciprofloxacin-coupled isatin analogues (**23a**–**h**, Figure 4a). The prepared conjugates were evaluated on their anti-TB potential against *Mycobacterium smegmatis* and *Mycobacterium tuberculosis* (MTB) H37Rv strains. The preliminary results indicate that all hybrids (MIC: 12.5–100 μg/mL) were considerably active against *M. smegmatis*, but lower than that of the mother ciprofloxacin (CPFX) and the reference INH. The conjugates exhibited excellent inhibitory potential towards MTB H37Rv with MIC values ranging from 1.56 to 25 μg/mL. Amongst the reported analogues, **23h** has proven a promising MIC of 1.56 μg/mL, which is two-fold more active than the mother CPFX with MIC 3.12 μg/mL. From the SAR analysis it is revealed that conjugates with methyloxime possess lower activity as compared to the corresponding carbonyl molecules against MTB H37Rv.

In another study, Xu et al. [47], in 2017, reported novel substituted isatin-propylene-1H-1,2,3-triazole-4-methylene-moxifloxacin conjugates **(24a-l)**. The anti-TB activities were evaluated against drug-sensitive and multidrug-resistant *M. tuberculosis*. Results of in vitro study suggested that all prepared conjugates displayed promising inhibitory potential against tested strains with MIC values ranging from 0.05 to 2.0 µg/mL. Among this, the hybrid **24i** exhibited excellent anti-TB potential, which is 2–8 times more than the standard moxifloxacin and RIF against *M. tuberculosis* H37Rv, whereas it was 2 -> 2048 times more potent than moxifloxacin, RIF, and INH against MDR *M. tuberculosis* (Figure 4a). The cytotoxicity study suggested that all synthesized analogues were found to be much more toxic than the mother moxifloxacin with CC_50_ 128 µg/mL against the VERO cell line.

Xu et al. [48], in their research conducted in 2017, assessed the anti-TB activity of 1H-1,2,3-triazole-tethered 8-OMe ciprofloxacin (8-OMe CPFX) isatin conjugates (**25a**–**l**, Figure 4a). The designed hybrids were evaluated for antimycobacterial activity against MTB, H37Rv, and MD-TB. A significant number of analogues have shown good inhibitory potential with MIC values in the range of 0.20–8.0 μg/mL against both MTB H37Rv and MDR-TB. Among those, hybrid **25h** has been found to be an excellent inhibitor of MTB H37Rv with MIC 0.20 μg/mL, which is 2–16 times more than the standards CPFX (MIC: 3.12 μg/mL), 8-OMe CPFX (MIC 1.56 μg/mL), and RIF (MIC 0.39 μg/mL). Meanwhile, as compound **25l** displayed potential towards MDR-TB with MIC 0.25 μg/mL, which is higher as compared to all reference drugs with an MIC range of 2.0–64 μg/mL. SAR indicated that the substation at the C-5 position of the isatin motif and iminies/carbonyl of Schiff’s base greatly influence the activity against both tested strains. Incorporation of -F at the C-5 position of isatin elevates the potency while the incorporation of electron-donating species causes the activity against both MTB H37Rv and MDR-TB strains to be lost.

In another study, Xu et al. [49], in 2017, developed a new set of ciprofloxacin-tagged isatin-1H-1,2,3-triazole analogues (**26a**–**l**, Figure 4a) for their antimycobacterial potential against MTB H37Rv, and cytotoxicity was measured for the VERO cell line. The obtained results indicate that all prepared hybrids are promising inhibitors of MTB H37Rv with MIC values ranging 0.39–50 μg/mL, while six of them were found to be the most active with MIC values ranging 0.39–1.56 μg/mL. Compound **26h** with MIC 0.39 μg/mL was found to be a highly active analogue compared to the reference RIF and CPFX. Evaluation against VERO cell lines suggested that all hybrids were more toxic with CC_50_ 4–64 μg/mL. Xu et al. [50] in 2018 designed a new series of homonuclear and heteronuclear bis-isatin hybrids tethered via ethylene (**27a**–**l**, Figure 4a), and their anti-TB potential was evaluated against MTB H37Rv and MDR-TB strains. All furnished hybrids have shown promising potential against both tested strains with MIC ranging from 16 to 256 μg/mL. In contrast, the heteronuclear bis-isatin derivative **27i** exhibited excellent potency with MIC values 25 to 16 μg/mL against both MTB H37Rv and MDR-TB, respectively. SAR analysis indicated that substitution of NNHCSNH_2_ at C-3 and -F at C-5 positions of the isatin motif was ideal for anti-TB potential. Overall, the mono-substituted bis-isatin derivative favored the activity compared to the bis-substituted and unsubstituted hybrids, while heteronuclear bis-isatin was proven to be more promising than the corresponding homonuclear analogues.

In 2018, Zhao et al. [51] studied the antimycobacterial activity of new tetraethylene glycol-tethered heteronuclear bis-isatin conjugates (**28a**–**l**, Figure 4b) against MTB H37Rv and MDR-TB as well as cytotoxicity in the VERO cell line. The majority of hybrids have shown potential to inhibit tested strains, in particular heteronuclear bis-isatin **28l** has shown the highest potency toward both MTB H37Rv and MDR-TB with MIC values of 16 and 16 μg/mL, respectively, which is more compared to the references RIF and INH. The active analogues have shown an acceptable cytotoxicity profile with CC_50_ 62.5 μg/mL. The SAR analysis explained that substitution at the C-3 position in the order of –NNHCONH_2_ > –NOMe > –NOEt > –O > –NOH influences activity, whereas the introduction of -F at the C-5 position improves the potency, and the electron-donating species -OMe diminishes it.

Gao et al. [52], in 2018, analyzed the anti-TB potential of novel benzofuran-isatin hybrids (**29a**–**i**, Figure 4b) against drug-susceptible MTB H37Rv, and MDR-TB as well as cytotoxicity were tested in VERO cells. The research team suggested that the conjugates displayed significant anti-mycobacterial potential with MIC values ranging from 0.25 to 8 µg/mL against both MTB H37Rv and MDR-TB with acceptable cytotoxicity in VERO cells with CC_50_ 128- >1024 µg/mL. Among this, compound **29i** displayed the most promising potential with MIC 0.25 and 0.5 µg/mL against MTB H37Rv and MDR-TB, respectively, which is better than that of the references RIF and INH. The hybrid **29i** was further subjected to access the metabolic stability and in vivo pharmacokinetic profile, and the obtained results indicated that it shows lesser metabolic stability as compared to the inhibitor TAM16. SAR analysis demonstrates that both C-3 and C-5 positions of the isatin ring significantly influence the activity. Compounds with a substitution of electron-donating species such as O-Me at the C-5 position could exhibit more potential when compared to -Br-substituted or -unsubstituted analogues. Hydrogen bond donor species such as hydrazinecarbothioamide or oximine could boost the anti-mycobacterial potential.

Novel propylene-tethered heteronuclear bis-isatin analogues (**30a**–**j**, Figure 4b) were reported for their anti-TB potential against MTB H37Rv and MDR-TB by Hua et al. [53] in 2018. The research group emphasized that all synthesized hybrids have significant potential of inhibition against both tested MTB H37Rv and MDR-TB strains with minimum inhibitory concentrations ranging from 16 to 256 μg/mL. Precisely, the heteronuclear bis-isatin hybrid **30i** proven to be the most active candidate in the series and shows MIC values of 25 and 16 μg/mL against MTB H37Rv and MDR-TB strains, respectively. The active analogue **30i** has shown four- and eight-fold more potency compared to the reference drugs RIF (64 μg/mL) and INH (>128 μg/mL) towards MDR-TB. SAR studies have concluded that the isatin moiety substituted at C-3 and C-5 positions could significantly influence the potency. Mono-substituted species at the C-3 position of bis-isatin analogues were found to be more active than the corresponding bis-substituted analogues.

A series of novel ethylene-tethered Isatin-Coumarin conjugates (**31a**–**j**, Figure 4b) were prepared by Gao et al. [54] in 2018. The prepared hybrids were screened for their in vitro antimycobacterial potential against MTB H37Rv and MDR-TB. The obtained results described that all isatin–coumarin hybrids were found to be active against both tested strains with MIC values ranging from 32 to 256 μg/mL. Among them, analogue **31h** has displayed extraordinary potential towards MTB H37Rv and MDR-TB with MIC values of 50 and 32 μg/mL, respectively, which was 2- and >4-fold more than the standard RIF and INH. SAR revealed that 5-flurosubstituted isatins have more potency than unsubstituted isatin moieties. Substitution at the C-3 position of the isatin motif greatly influences the activity against MTB H37Rv and MDR-TB.

In 2018, Hao-Di Wang and co-workers [55] reported the antimycobacterial potential of propylene-tethered gatifloxacin-isatin conjugates (**32a**–**f**, Figure 4b) against MTB H37Rv and MDR-TB as well as cytotoxicity against the VERO cell line. The preliminary screening emphasized that the entire furnished conjugates displayed promising potential with MIC values ranging from 0.25 to 16 μg/mL against both tested strains with acceptable cytotoxicity (CC_50_ 32–512 μg/mL). The hybrid **32e** from mono-isatin gatifloxacin was proven to be the most active analogue in the series with MICs of 0.25 and 0.25 μg/mL against MTB H37Rv and MDR-TB strains. The most active candidate **32e** exhibited two-fold more potency compared to the mother gatifloxacin and RIF (MIC 0.5 and 0.25 μg/mL) against MTB H37Rv and 4- to >512-fold more compared to gatifloxacin, RIF, and INH against MDR-TB. SAR analyses found that mono-isatin-gatifloxacin derivatives were more promising as compared to the corresponding bis–isatin–gatifloxacin hybrids. The incorporation of methoxyimino at the C-3 position and electron-withdrawing species -F at the C-5 position of the isatin moiety enhances the anti-TB potential.

In another study accomplished in 2018, Yan Xu et al. [56] investigated the antimycobacterial activity of isatin-(thio) semicarbazide/oxime-1H-1,2,3-triazole-coumarin conjugates (**33a**–**l**, Figure 4b) towards the MTB H37Rv and MDR-TB strains. The furnished results illustrate that all hybrids displayed weak to moderate potential with MIC values ranging from 50–>200 μg/mL, which is much less than the reference INH and RIF. In the series, compound **33h** was found to be active against MTB H37Rv and MDR-TB strains with MIC 50 μg/mL, which is more compared to isoniazid (MIC > 128 μg/mL) against MDR-TB. In 2018, Deng et al. [57] proposed antimycobacterial activity of heteronuclear 5-fluoroisatin dimers (**34a**–**j**, Figure 4b) against MTB H37Rv and MDR-TB strains. All synthesized conjugates have shown significant potential towards the two tested strains with MIC ranging from 25 to 256 μg/mL. In contrast, the heteronuclear 5-fluoroisatin dimer **34a** exhibited the most promising inhibitory activity towards H37Rv and MDR-TB strains with MIC values of 25 to 32 μg/mL, respectively. The active analogue **34a** displayed two-fold greater potency as compared to RIF (MIC 64 μg/mL) and INH (MIC >128 μg/mL) towards MDR-TB.

Wang et al. [58] in 2018 reported the antibacterial and anti-TB potential of some propylene-tethered ciprofloxacin-isatin hybrids (**35a**–**f**, Figure 4b). The prepared analogues were tested for their antimycobacterial potential, cytotoxicity in VERO cell lines, metabolic stability, and in vivo pharmacokinetic profile. The preliminary testing indicated that most of the hybrids have shown considerable potency, and in particular, hybrid **35b** has shown excellent antitubercular potential with MICs of 0.10 and 0.5 mg/mL against MTB H37Rv and MDR-TB, respectively. The active hybrid **35b** shows 4- and 8-fold more activity than that of the mother CPFX (MIC: 0.78 mg/mL) and RIF MIC 0.39 mg/mL against MTB H37Rv, while it shows 4 to > 256 times more activity than the standards CPFX, RIF, and INH with MICs of 2.0, 32, and >128 mg/mL, respectively. The active analog exhibited adequate cytotoxicity, metabolic stability, and a PK profile. SAR validated that mono-isatin-CPFX analogues displayed better potential compared to bis-isatin-CPFX candidates.

In 2019, Gao et al. [59] demonstrated in their another study the antimycobacterial and antibacterial potential of some novel benzofuran-isatin-imine conjugate links via propylene, butylene, pentylene, and hexylene (**36a**–**v**, Figure 4c). All prepared conjugates have shown significant activity, with MIC values ranging between <0.016 and 0.218 µg/mL and 0.062 and 14.15 µg/mL towards MTB H37Rv and MDR-TB, respectively, along with adequate cytotoxicity towards VERO cells (CC_50_ 8–128 mg/mL). The most active analogue from the series, hybrid **36j,** has proven to be excellent towards MTB H37Rv and MDR-TB isolates with MIC values of 0.016, 0.062, and 0.16 mg/mL, respectively. It was <4.8- and <48-fold higher as compared to the standard drugs RIF and INH. SAR analysis indicated that the incorporation of imines at the C-3 position of the isatin ring and -F at the *para* position of phenyl has resulted in the enhanced cytotoxicity profile.

Another research work by Gao et al. [60] in 2019 illustrated the anti-TB activity of benzofuran-isatin analogues tethered via alkyl linkers (**37a**–**u**, Figure 4c) against two MDR-TB isolates and evaluated the cytotoxicity towards CHO cells. The primary screening determined that the majority of the candidates displayed promising activity against the tested MDR-TB strains with MIC values ranging between 0.125 and 16 mg/mL with acceptable cytotoxicity (CC_50_ 64- >512 mg/mL). From the series, conjugate **37s** exhibited promising anti-TB potential with MIC values of 0.125 and 0.25 mg/mL against both tested MDR-MTB strains, which is 8–16-fold more than the standard CPFX and 512-fold greater than RIF and INH. The anti-TB activity was found to be closely dependent on the substitution at C-3 and C-5 positions of the isatin motif. The C-3 position, which engaged with thiosemicarbazide and methyloxime, accomplished better potency than the corresponding ketone derivatives, whereas hydroxylamine was unfavorable for activity. The C-5 position of the isatin nucleus, when substituted by an electron-withdrawing group, increases activity while OMe decreases and reduces the potential as compared to the unsubstituted conjugates. The longer linker was also advantageous for anti-TB potential.

In 2019, Gao et al. [61], in another study, explained the synthesis and anti-tubercular activity of fourteen moxifloxacin-acetyl-1,2,3-1H-triazole-methyleneisatin analogous (**38a**–**n**, Figure 4c) against drug-susceptible MTB H37Rv and MDR-TB as well as cytotoxicity towards VERO cells and inhibitory potential towards MTB DNA gyrase. The research group highlighted that compounds **38h** and **38l** were found to be the most active analogues in the series with MIC values of 0.12 and 0.5 mg/mL, respectively, against all tubercular strains. SAR and SCR demonstrate that the C-3, C-5, and C-7 positions of the isatin molecule can significantly influence the anti-TB potential and cytotoxicity against the tested strains. Incorporation of the thiosemicarbazone group at the C-3 position of the isatin moiety greatly enhances the potency, whereas the substitution of oxime and methyloxime has a detrimental effect on activity when compared with ketone. Analogues with electron-withdrawing and -donating substitutions at the C-5 position of the isatin motif have shown a loss in potential when compared to the unsubstituted analogues. While the structure—cytotoxicity relationship revealed the isatin moiety substituted at the C-3 position by oxime and thiosemicarbazone and -F at either C-5 or C-7 positions may enhance the cytotoxicity, methyl at C-5 and methyloxime at the C-3 position of the isatin nucleus could lead to a loss of cytotoxicity.

Series of novel amide-linked ciprofloxacin-1,2,3-triazole-isatin conjugates (**39a**–**l**, Figure 4c) were furnished by Chen et al. [62] in 2019, and their in vitro antimycobacterial potential on MTB H37Rv and MDR-MTB strains and cytotoxicity in VERO cells were evaluated. The research team highlighted that the prepared conjugates possess significant anti-TB potential against tested strains with considerable cytotoxicity in MIC ranges from 0.12 to 32 μg/ mL and CC_50_ 8.0–>128.0 μg/mL. Compound **39a** was proven to be the most active analogue from the series with MIC 0.5 μg/mL against MTB H37Rv and 0.12 μg/mL towards MDR-MTB, whereas compound **39a** displayed acceptable cytotoxicity towards VERO cells with CC_50_ 16.0 μg/mL. A series of butylene-tethered 7-fluoroisatin-isatin hybrids (**40a**–**h**, Figure 4c) were designed and synthesized by Liu et al. [63] for their anti-TB potential in 2019. The resultant heteronuclear analogues were screened for their anti-TB potential against MTB H37Rv and MDR-MTB strains. Among the series, compound **40e** was found to be highly active against MTB H37Rv and MDR-MTB with MIC values of 1 and 4 μg/mL, respectively. Compound **40e** was found to be ≥16-fold more potent than the standard INH, RIF, and EMB against the MDR-MTB strain. Moreover, heteronuclear **40e** also displayed an excellent toxicological profile as well as excellent inhibitory potential towards MTB DNA gyrase. A SAR study highlighted that the substituted C-3 position of isatin and 7-fluroisatin greatly influences the potential. Analogues bearing different substitutions were found highly active as compared to the scaffold with the same substitution at both isatin moieties.

Ding and co-workers [64] in 2019 reported the antimycobacterial potential of a series of gatifloxacin-1,2,3-triazole-isatin conjugates (**41a**–**l**, Figure 4c) and cytotoxicity towards CHO cells. The research group emphasized that all prepared hybrids have displayed significant inhibitory activity against MTB H37Rv and two MDR-MTB strains with MIC values ranging between 0.25 and8 μg/mL with considerable cytotoxicity. In contrast, compound **41a** exhibited promising anti-TB potential with MIC 0.25 μg/mL against MTB H37Rv and 0.5 and 1 μg/mL towards two MDR-MTB strains. Along with that, compound **41a** (CC_50_ 8.0 μg/mL) also displayed considerable cytotoxicity towards CHO cells. The incorporation of imine by replacing ketone at the C-3 position of the isatin moiety cannot increase the potency, while the C-5 position was very important for potential. The electron-withdrawing -F group improves the potency while electron-donating methyl decrease the potential. Recently, Elsayed et al. [65] screened the anti-TB and antibacterial potency of novel isatin-nicotinohydrazide hybrids (**42a**–**m** and **43a**–**c**, Figure 4c). The anti-TB activities were evaluated against the drug-susceptible MTB strain (ATCC 27294) and INH/Streptomycin-resistant MTB (ATCC 35823). Among the series, analogues **42d**, **42g**, and **42h** displayed excellent potential against MTB (ATCC 27294) with MIC 0.24 μg/mL, while compounds **42g** and **42h** exhibited potent inhibitory potential with MIC 3.9 mg/mL against MTB (ATCC 35823). The active analogues were further subjected to docking simulation to determine the hydrogen bonding interaction within the cavity of decaprenylphosphoryl-b-D-Ribose 20-Epimerase (DprE1). It was proposed that compounds **42g** and **42h** show the best energy scores, at 10.1 and 9.7 kcal/mole, and the H-bond between carbonyl C=O with amino acid residues His-132 and Tyr-415 and hydrazide NH as well as the N-atom of pyridine displayed H-bond interactions with His-132 and Asn-385, respectively [65]. 

### 2.3. Isatin as Antimicrobial Agent

**Figure 5:**
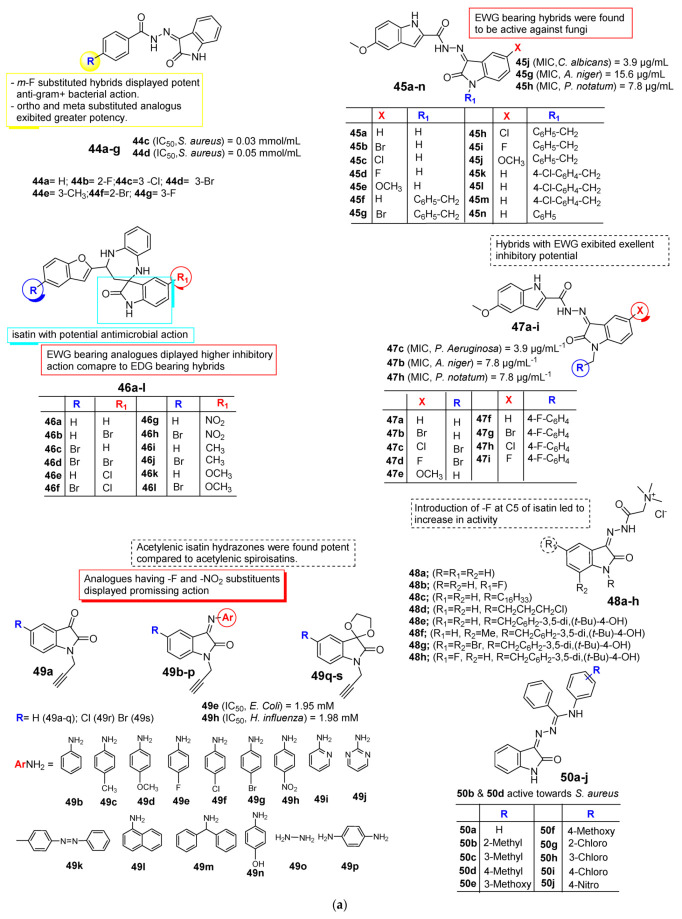

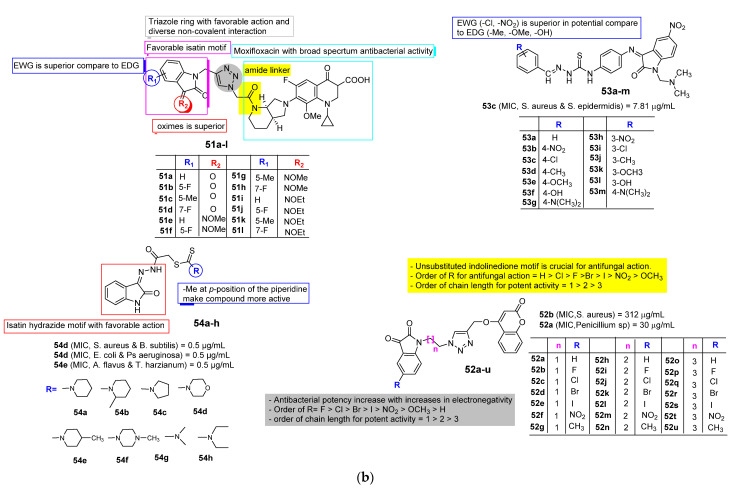
(**a**) SAR features contributing to antimicrobial potential of some isatin analogues. (**b**) SAR features contributing to antimicrobial potential of some isatin analogues.

In 2016, Lian et al. [66] reported the antibacterial activity of some novel isatin analogues (**44a**–**g**, Figure 5a) as FtsZ inhibitors. The antibacterial potential of the reported molecules was screened against *B. subtilis*, *E. coli*, *P. aeruginosa*, and *S. aureus*. The majority of analogues exhibited potent antibacterial inhibitory potential against the tested microorganism. Compounds **44c** and **44d** displayed promising activity against *Staphylococcus aureus* with IC_50_ values of 0.03 and 0.05 mmol/mL, respectively, while analogue **44g** exhibited better potential against *Escherichia coli* and *Pseudomonas aeruginosa* with IC_50_ values of 0.672 and 0.830 mmol/mL. A molecular docking study shows the prepared isatin analogues were fit to the binding pocket of FtsZ. In contrast, compounds **44b** and **44d** show hydrogen bond interactions with six amino acids along with a p-cation interaction with Arg143, while analogue **44g** displayed H-bond interactions with four amino acid residues as well as p-cation interactions with Arg 143 [66].

A study published by Al-Wabli et al. [67] in 2017 described the antibacterial activity of some novel isatin–indole molecular hybrids (**45a**–**n**). The prepared derivatives were screened for their in vitro antimicrobial potential against a panel of *P. aeruginosa*, *E. coli*, *K. pneumoniae*, *P. vulgaris*, *S. enteridis*, *E. fecalis*, *S. aureus*, *B. subtilis*, MRSA bacteria as well as *P. notatum*, *A. niger*, and *C. albicans* fungal strains via the disk diffusion assay method. Most of these derivatives showed weak to no activity against most of the tested microorganisms, while analogues **45b** and **45c** show significant activity against Gram +ve bacteria. In contrast, compound **45j** was found to be the most active candidate against *C. albicans* with an MIC value of 3.9 µg/mL, which was four times more potent than the reference FLC, while compound **45g** exhibited promising activity with MIC 15.6 µg/mL towards *A. niger*. On the other hand, compound **45h** displayed potent antifungal activity against *P. notatum* with an MIC value of 7.8 µg/mL (Figure 5a).

Kenchappa et al. [68] in 2017 screened fused isatin and diazepine analogues (**46a**–**l**) against a panel of bacterial and fungal strains using the agar well diffusion method. From the established analogues, compounds **46d** and **46f,** with electron-withdrawing species at the C-8 position of benzofuran and the isatin motif, exhibited the highest potential against all tested bacterial strains with MIC values between 0.20 and 0.30 mg/mL. Analogue **46e,** having Cl at the C-8 position of isatin, and **45h,** having Cl and NO_2_ at the C-8 position of benzofuran and the isatin motif, respectively, display the highest potency towards *B. subtilis* and *E. coli*. From the obtained results, it was noted that unsubstituted benzofuran and the isatin moiety lowered the potency of the analogues. On the other hand, analogues **46e** and **45h,** with Cl and Br, at NO_2_, showed promising antifungal activity against *A. flavus* and *C. albicans*, while compounds **46b** and **46c,** with a substitution of Br, displayed promising activity towards *M. griseous*, *A. flavus,* and *M. griseous*. In 2017, Almutairi et al. [69] investigated the in vitro antimicrobial activity of new indole-isatin molecular hybrids (**47a**–**i**, Figure 5a) against a panel of Gram-positive and Gram-negative bacteria and molds via disk diffusion assay. The research team emphasized that all synthesized hybrids were found to be inactive against Gram-negative pathogens when compared to the reference drug, except *Ps. aeruginosa*. The synthesized hybrids were found to be promising towards Gram +ve strains compared to the Gram -ve and have shown high potency towards the tested filamentous fungi. Among the series, compound **47c** appeared to be the most active analogue from the series with MIC 3.9 µg mL^−1^ against *Ps. Aeruginosa,* eight-fold more than that of standard AMP, while hybris **47i** exhibited promising activity against *A. niger* and compounds **47b** and **47h** displayed equipotent activity against *P. notatum* with MIC values of 7.8 µg mL^−1^.

Novel water-soluble isatin-3-acylhydrazones **(48a**–**h**, Figure 5a) were prepared and screened for their in vitro antimicrobial potential against Gram +ve bacterial and fungal strains in 2017 by Bogdanov et al. [70]. Among the synthesized isatin-3-acylhydrazones, **48b**, **48f**, **48g**, and **48h** exhibited good to moderate potential towards *S. aureus*, *B. cereus,* and *C. albicans*. Compound **48e** was found to be 16-fold more active than standard chloramphenicol against *S. aureus* and 4 times than *B. Cereus*. SAR analysis revealed that the incorporation of fluorine at the C-5 position of the heterocycle enhances the potency of molecules compared to unsubstituted molecules. Acylhydrazone with a fluoro group displayed two times greater potency against *B. cereus* as compared to the reference/standard chloramphenicol.

Singh et al. [71], in 2018, reported the in vitro antimicrobial activity of novel acetylenic isatin hydrazone (**49b**–**p**) and acetylenic spiroisatins (**49q**–**s**) against bacterial and fungal strains, and the obtained results were compared to reference drugs with kanamycin and amphotericin B, respectively. The obtained results revealed that all prepared acetylenic isatin displayed remarkable antimicrobial potential. Compound **49e,** having -F substituents, displayed eminent potency with an IC_50_ value of 1.95 mM towards *E. coli*, while analogues **49e** and **49h,** having -F and -NO_2_ substitutions, respectively, exhibited the most promising activity towards *S. aureus*. Meanwhile, compound **49e** only shows admirable potential towards *E. faecalis* with an IC_50_ value of 7.81 mM. Moreover, compound **49h** with -NO_2_ exhibited an exemplary effect with an IC_50_ value of 1.98 mM against *H. influenza*. Overall, the obtained results revealed acetylenic isatin hydrazone displayed the most promising potential towards tested bacterial strains compared to acetylenic spiroisatins. Regarding the antifungal potential, the bis-acetylinic compound **49n** presented the most eminent activity towards *C. albicans* with an IC_50_ value of 15.67 mM, while compound **49m** with benzhydryl substitution exhibited outstanding potential towards *C. parapsolsis* fungi (Figure 5a).

In 2019, Gomaa et al. [72] reported the synthesis and in vitro antimicrobial potential of N-aryl-N′-(2-oxoindolin-3-ylidene)-benzohydrazonamides (**50a**–**j**) against *B. cereus*, *S. aureus* (Gram-positive), and *E. coli*, *P. aeruginosa*, and *S. marcescens* strains using the agar disc diffusion method. The research group emphasized that compounds **50b** and **50d** show the most promising activity towards *S. aureus* when compared to the standard drug ciprofloxacin, while the majority of analogues displayed broad-spectrum antibacterial potential against all tested pathogens compared with ciprofloxacin (Figure 5a).

Novel moxifloxacin-amide-1,2,3-triazole-isatin conjugates (**51a**–**l**, Figure 5b) were designed and synthesized for their antibacterial potential by Gao et al. [73] in 2019. The prepared hybrids have shown significant antibacterial potential against tested pathogens with MIC values ranging from ≤0.03 to 128 μg/mL. Compounds **51e**, **51g**, and **51j** displayed promising potential against the tested strains compared to the parent moxifloxacin (MIC ≤ 0.03–8 μg/mL). Moreover, the promising analogues **51e**, **51g**, and **51j** proved to be less cytotoxic towards CHO cells. SAR stated that the substituents at the R1 position greatly affect the potency, while electron-donating species (methyl) have greater potential than electron withdrawing (fluoro). The substituents on the C-5 position exhibited greater activity than the C-7 position of hybrids. The C-3 position of the isatin nucleus substituted with methyloxime could enhance the potential, while ethyloxime is generally detrimental to antibacterial potential.

Bhagat et al. [74] in 2019 developed a new series of indolindione−coumarin molecular hybrids (**52a**–**u**, Figure 5b) for their in vitro antimicrobial potential against *E. coli*, *S. enterica*, *S. aureus*, *M. smegmatis*, *C. albicans*, *A. mali*, *Penicillium* sp., and *F. oxysporum* using the agar gel diffusion method. The biological activity study revealed that all the hybrids are active against tested bacterial strains. Among the hybrids **52b** was found to be the most active analogue in the series against *S. aureus* with MIC 312 μg/mL, while compound **52a** displayed promising potency with MIC 30 μg/mL towards *Penicillium sp*. SAR analysis demonstrates that the length of the carbon chain between triazole and indolinedione frameworks as well as the electronic environment in the indolinedione moiety greatly distress the activity of the newly synthesized hybrids.

In 2020, Lahari et al. [75] designed a new set of indole-2,3-dione analogues **(53a**–**m)** for their analgesic, anti-inflammatory, and antimicrobial potential. The antimicrobial potential of furnished analogues was examined against a panel of bacterial and fungal strains. The obtained results revealed that compounds **53b**, **53c**, **53h**, and **53i** displayed promising antimicrobial potential with MIC 7.81 mg/mL which is better than the reference ciprofloxacin against *S. aureus* and *S. epidermidis*. In contrast, the derivative 1-(4-nitrobenzylidene)- 4-(4-(1-((dimethylamino)methyl)-5-nitro-2-oxoindolin3-ylideneamino) phenyl) thiosemicarbazide **53b** was proven to better towards *M. leutus*, whereas it exhibited equipotent antimicrobial potential against *P. aeruginosa*, *E. coli*, *S. epidermidis,* and *A. fumigatus,* like the reference candidate. SAR established that the compound bearing electron-withdrawing species such as chloro and nitro groups displayed potent antibacterial and antifungal action while the remaining analogues from the series, with electron-donating methyl, methoxy, hydroxyl, or dimethylamino species, found less potential compared to the reference analogues (Figure 5b). In 2020, Mangasuli [76] developed isatin-dithiocarbamate conjugates (**54a**–**h**) and screened for their antimicrobial potential against *S. aureus*, *B. subtilis*, *E. coli*, and *P. aeruginosa* bacterial strains and *A. flavus*, *T. harzianum*, *P. chrysogenum*, and *C. albicans* fungal strains, and compared the obtained results with the reference drugs ciprofloxacin and fluconazole. The obtained results exposed that most candidates were found to be active against all tested bacterial strains. The hybrid analogue **54d** was found to be the most active analogue against *S. aureus* and *B. subtilis* with MIC 0.5 μg/mL, whereas compound **54a** was found to possess significant activity against *S. aureus*, *B. subtilis,* and *E. coli* with MIC of 1 μg/mL (Figure 5b). The in vitro antifungal activity study revealed compound 54e exhibited the most promising activity with an MIC value of 0.5 μg/mL towards *A. flavus* and *T. harzianum,* which is less than the reference fluconazole. Additionally, compounds **54b, 54c**, and **54d** displayed very good potential against *C. albicans* with an MIC value of 1μg/mL. Essential structural features for potent antimicrobial activity are illustrated in Table 1.

### 2.4. Isatin as Anti-Convulsant Agent

**Figure 6:**
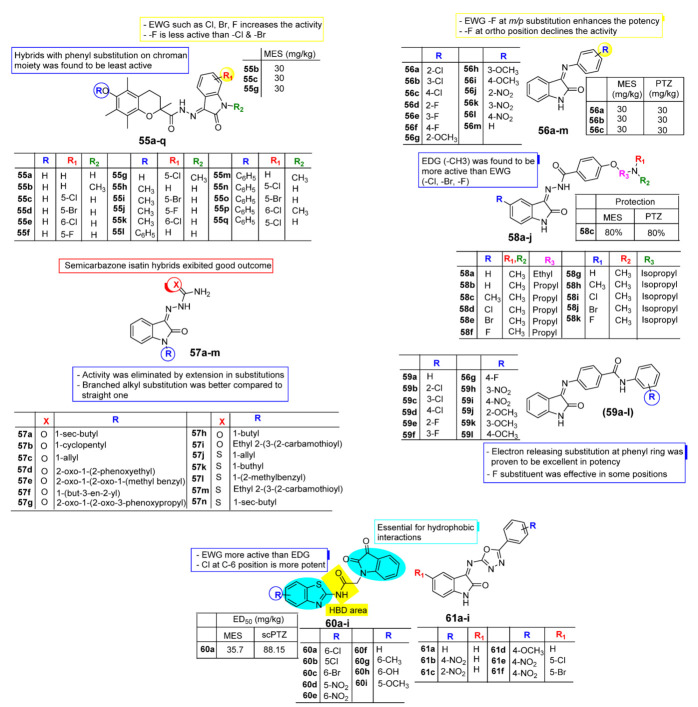
SAR features suggesting anticonvulsant potential of isatin derivatives.

In 2016, Rawat et al. [77] developed a series of novel chroman-fused isatin analogues (**55a**–**q**, Figure 6) for their anti-breast-cancer and antiepileptic potential. The anticonvulsant screening was assessed via MES and PTZ seizures, and neurotoxicity was examined using a rotarod test. The researchers underlined that those analogues with a hydroxyl group on the chroman ring, such as **55b**, **55c**, **55d**, **55e**, and **55g,** exhibited more anticonvulsant potential compared to the hybrids with a methyl substitution such as **55h**, **55i**, and **55k**. Analogues with a phenyl substitution on the chroman moiety were found to be the least active. Compounds with electron-withdrawing substituents Cl, Br, and F on the isatin nucleus might have enhanced the potential compared to the analogues with electron-donating species, while compounds substituted with -F were found to be less active than the compounds with Cl and Br substitutions. None of the prepared analogues have shown neurotoxicity as detected by the rotarod test.

Rahmani-Khajouei et al. [78], in 2017, synthesized some isatin-based derivatives (**56a**–**m**, Figure 6) for their anticonvulsant potential using MES and PTZ, and a neurotoxicity study assessed this using a rotarod test. Among the newly prepared analogues, the majority of the compounds displayed noteworthy preventive outcomes at a 30 mg/kg dose in both MES and PTZ models with acceptable neurotoxicity. A SAR study revealed that the compound with electron-withdrawing species -F caused an increased in potential compared to -Cl. The position of the substitution of -F greatly influences the activity. F incorporated at *meta* and *para* positions displayed protection in both models, while -F in an *ortho* position decreased the potential. Chlorinated analogues **56a**, **56b**, and **56c** exhibited prevention at a dose of 30 mg/Kg in both MES and PTZ. Analogues with nitro substitution such as **56j**, **56k,** and **56l** were found to be active analogues at a dose of 30 mg/kg, especially in the MES model. Moreover, substitution of the nitro group at *ortho* and *para* positions also showed good prevention in the PTZ model. Analogues with electron-donating substitutions did not show satisfactory anticonvulsant potential at 30–300 mg/Kg doses. Perhaps the hydrophilic nature and electron-donating property of the molecules do not have good factors to improve anticonvulsant potential. Fortunately, none of the tested analogues showed neurotoxicity at 30 mg/kg in the rotarod model. A set of semicarbazone and thiosemicarbazone analogues of isatin (**57a**–**n**, Figure 6) were synthesized for their anticonvulsant potential by Khabnadideh et al. [79] in 2017. The antiseizure screenings were assessed via the PTZ model in mice and a chemical kindling model in male rats. Compounds **57b**, **57d**, **57f**, **57i**, **57j**, **57k**, **57l**, **57m**, and **57n** were found to be significantly active in PTZ screening at three dose levels, namely 10, 20, and 30 mg/kg. On the other hand, analogues **57k** and **57n** were proven to be effective in the kindling model at 10 and 30 mg/kg doses. All the active analogues from the above set of semicarbazone and thiosemicarbazone isatin improved motor coordination in the rotarod test. A SAR study proposed that among the semicarbazone isatin analogues, N-substituted derivatives exhibited a good outcome, while the potential was eliminated by the extension of substitutions. This may have happened due to an increase in molecular weight as a result of the incorporation of a large group. In the case of compounds **57k** and **57n,** it seems that the branched alkyl substitution was better than the straight one regarding solubility.

Madhira et al. [80], in 2017, reported the anticonvulsant potential of some novel N, N-dialkylaminoalkoxy-2-Oxo-indole-3-ylidene benzohydrazides (**58a**–**k**). MES- and PTZ-induced seizure models are used to evaluate the anticonvulsant activity of the prepared set of analogues. Compound N, N-dimethylamino propoxy-2-Oxo-5-methyl-3-ylidenebenzo hydrazide **58c** (-CH_3_) shows 80% protection in both MES and PTZ models, whereas compounds **58e**, **58d**, **58b**, and **58f** were found to be the next active in the duration of convulsions in both MES and PTZ. Compounds with electron-releasing substitutions (−CH_3_) at the C-5 position of the isatin motif formed 80% protection in both MES and PTZ, while analogues with an electron-withdrawing substitution, such as -Cl, -Br, and -F at the C-5 position of the isatin moiety, were observed to be next in the order of potential (Figure 6). Rahmani-Khajouei et al. [81], in their research carried out in 2018, illustrated the antiepileptic activity of (Z)-4-(2- oxoindolin-3-ylideneamino)-N-phenyl benzamide analogous (**59a**–**l**). These newly synthesized analogues were assessed in terms of their anticonvulsant and neurotoxic properties. The majority of the tested analogues showed significant protection with good safety levels in the neurotoxicity test. in contrast, compounds **59j**, **59k**, and **59l** exhibited promising antiepileptic potential in the MES model while analogue **59j** with 2-OCH_3_ and **59l** with 4-OCH_3_ were proven to be excellent in the PTZ model. The active analogues demonstrate high safety regarding neurotoxicity at a dose of 30mg/kg, except **59l** with 4-OCH_3_ (Figure 6).

In 2020, Nath et al. [82] designed and synthesized the indoline analogues of benzothiazole acetamide (**60a**–**i**) and aryloxadiazole amine (**61a**–**f**) for their antiseizure activity using MES and scPTZ models, and neurotoxicity was assessed via the motor impairment mode. Analogue **60a** i.e., N-(5- chlorobenzo[d]thiazol-2-yl)-2-(2,3-dioxoindolin-1-yl) acetamide emerged as the most promising analogue from a set of furnished derivatives against both MES (with ED_50_ 35.7 mg/kg) and scPTZ (with ED_50_ 88.15 mg/kg) models, having TD_50_: >500 mg/kg. The aryloxadiazole amine series (**61a**–**f**) was found to be toxic at the maximum dose. SAR analysis demonstrated that the substituted C-5 and C-6 positions of the benzothiazole framework greatly impact the potency of indoline-fused benzothiazole analogues. In general, the compound substituted with halogen species exhibited greater potential as compared to the analogues with electron-withdrawing or electron-donating groups (Figure 6). Compound **60a** with a substituted C-6 position of benzothiazole by *para*-chloro displayed greater activity, whereas it resulted in decreased potential when chloro was incorporated at the C-5 position. The compound substituted with the bromo group at the C-6 position of benzothiazole was found active but remained associated with neurotoxicity. Incorporation of NO_2_ either at C-5 or C-6 revealed significant activity at a higher dose. Replacement of the electron-withdrawing groups with electron-donating groups such as CH_3_, -OCH_3_, and –OH resulted in loss of activity. Structural characteristics for anticonvulsant action are revealed in Table 1.

### 2.5. Antioxidant Potential of Isatin

**Figure 7:**
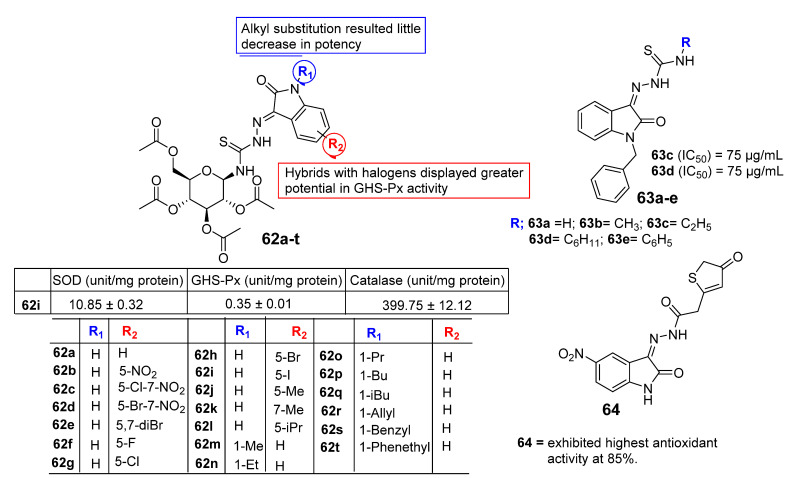
SAR features suggesting antioxidant activity of isatin derivatives.

New substituted isatin N-(2,3,4,6-tetra-O-acetyl-b-D-glucopyranosyl) thiosemicarbazones analogues (**62a**–**t**) were synthesized and evaluated for their antioxidant and in vitro antimicrobial activity by Thanh et al. [83] in 2016 (Figure 7). The antioxidant activity of the prepared compounds was evaluated by means of superoxide dismutase (SOD), glutathione peroxidase (GHS-Px), and catalase. The screening results have shown that analogues bearing halogen species except fluorine seem to be promising in SOD activity, and the MIC values are **62c** MIC 10.57 ± 0.31, **62d** MIC 10.76 ± 0.33, **62e** MIC 10.89 ± 0.25, **62g** MIC 10.20 ± 0.29, **62h** MIC 10.47 ± 0.19, and **62i** MIC 10.85 ± 0.32 unit/mg protein. The presence of an alkyl substitution at position-1 of the isatin motif resulted in a slight decrease in potential. On the other hand, the same condition appeared in the case of GHS-Px and catalase activity. Analogues with halogen exhibited greater potency and were found to be more active in GHS-Px activity with MIC values ranging between 0.27 and 0.93 units/mg protein compared to the control resveratrol, whereas the incorporation of an alkyl group at the nitrogen atom of isatin slightly enhances the potential. Among this, compound **62r** was the most potent with MIC 0.27 ± 0.01 units/mg protein in GHS-Px activity. Compounds **62c**–**i**, **m**–**r** displayed the highest catalase activity with MIC values ranging between 345.45 and 399.75 unit/mg protein. In contrast, analogue **62i** was found to be the best, with MIC 399.75 ± 12.12 units/mg.

In 2016, Haribabu et al. [84] investigated the antioxidant potential of Isatin-based thiosemicarbazone analogues (**63a**–**e**). The synthesized compounds were evaluated for their antioxidant activity using a DPPH scavenging assay. Compounds **63c** and **63d** were found to be the most potent with IC_50_ values of 75 µg/mL, while the IC_50_ value of compound **63a** was found to be 250 µg/mL (Figure 7). In 2019, El-Serwy and their team [85] investigated the antioxidant, anticoagulant, and fibrinolytic activity of some new isatin analogues. Compound **64** displayed excellent antioxidant potential at 85%. The presence of an active methylene group in the compounds increased the antioxidant potential. 

### 2.6. Anti-Inflammatory Potential of Isatin

In 2016, Jarapula et al. [86] synthesized new 2-hydroxy-N′-(2-oxoindolin-3-ylidene) benzohydrazide analogues (**65a**–**j**) via the condensation of different substituted isatins with 2-hydroxybenzohydrazide. The prepared derivatives were evaluated for their anti-inflammatory activity by the carrageenan-induced paw edema technique using indomethacin as a positive control. The presented results emphasized that analogues **65c** and **65d** displayed the most promising potential with paw edema percent reductions of 65% and 63%, respectively. SAR analysis revealed that analogues **65c** (5-Cl), **65d** (5-Br), **65i** (7-Cl), and **65j** (7-Br) with halogen species at C-5 and C-7 positions of the isatin ring exhibited promising anti-inflammatory potential. Meanwhile, derivatives with alkyl and acetyl substitution at the N-1 position of the isatin framework were displayed next in the order of potential. Prepared analogues docked into the active site of COX-1 (PDB ID: 3N8Y) and COX-2 (PDB ID: 3LN1). Compounds **65c**, **65d**, and **65f** displayed good binding scores of 57.27, −62.02, and −58.18 kcal/mol with the binding pocket of COX-2 and the lowest dock scores of −8.03, −9.17, and −8.94 kcal/mol on COX-1 enzymes. Compounds **65c** and **65d** displayed an H-bond interaction with amino acid residues Gln-275 and His-193 while compound **65f** exhibited hydrophobic interactions with amino acid residues Lys197, Asn217, His218, Gly221, Gly274, Glu222, and Gln275 with the active site of COX-2 [86].

**Figure 8:**
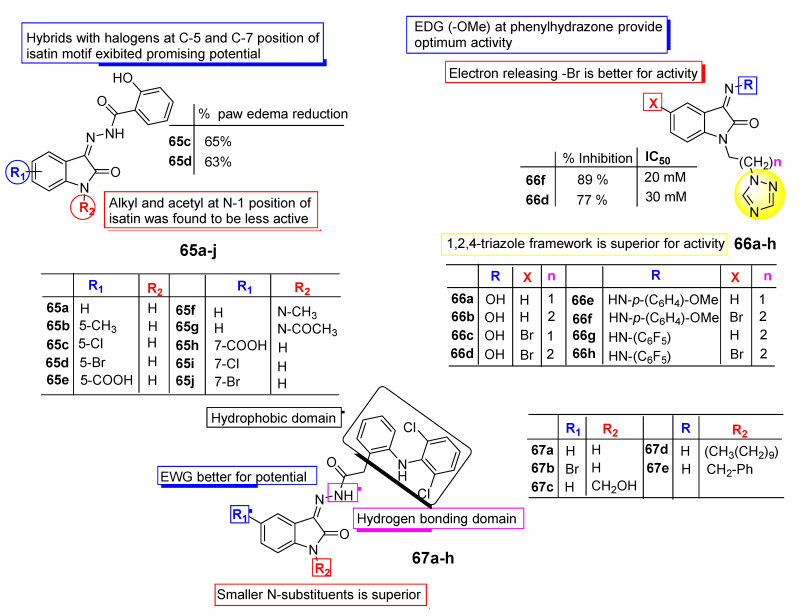
SAR features of isatin and its derivatives required for potent anti-inflammatory potential.

Novel triazolyl isatin conjugates (**66a**–**h**) were synthesized by Sharma et al. [87] in 2016. The prepared hybrids were checked for their anti-inflammatory activity with respect to the TNF-a-induced expression of ICAM-1 on the surface of human endothelial cells. The research group suggested that analogue **66f** demonstrated the most promising activity against ICAM-1 expression with a percent of inhibition of 89.00% with an IC_50_ value of 20 mM at a 200 mM maximal tolerable dose. Meanwhile, hybrid **66d** was found to be the second most active analogue in the series and displayed 77.00% maximum inhibition of ICAM-1 expression at a 150 mM dose with an IC_50_ value of 30 mM. Analogues with electron-releasing bromo species at the C-5 position of isatin species have greater potential; in addition, the incorporation of the 1,2,4-triazole framework and electron-donating methoxy group at the phenylhydrazone ring presented an increase in potential (Figure 8). A study conducted in 2018 by Ibrahim [88] reported the anti-inflammatory potential of novel, isatin-fused diclofenac conjugates (**67a**–**e**). Compounds **67a** and **67b** originated as the most active analogues from the prepared conjugates. Compound **67b** exhibited the most prominent in vivo anti-inflammatory potential (61.32% inhibition) compared to the positive control diclofenac (51.36% inhibition). SAR analyses demonstrate that the hydrogen bond acceptor is essential for the interaction with amino acid residues, while the aromatic framework in diclofenac is essential for hydrophobic interactions with hydrophobic amino acid residues in the binding pocket of receptors (Figure 8). Analogues bearing smaller substituents at the N-position of the isatin nucleus induced greater potential, while bulky N-substituents such as benzyl and decyl groups cause a prominent decline in binding to receptors. Docking analysis indicated compound **67b** had a docking score of −10.765 kj/mol and formed strong hydrogen bond interactions with Tyr385 and Ser530 and has proven to be a potent COX enzyme inhibitor. The substitution pattern is important for antioxidant potency [88]. 

### 2.7. Antidiabetic Profile of Isatin

**Figure 9:**
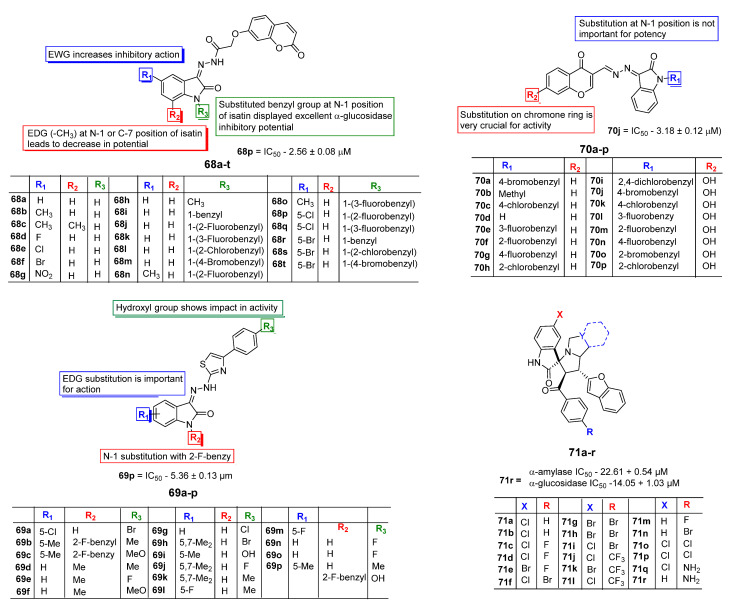
Chemical structures and SAR features for anti-diabetic profile of isatin conjugates.

To provide potential leads for diabetes treatment, Wang et al. [89] in 2016 synthesized coumarin-fused isatin derivatives (**68a**–**t**, Figure 9) for their α-glucosidase inhibitory potential. The majority of the analogues displayed promising inhibitory potential towards α-glucosidase with IC_50_ values ranging 2.56 ± 0.08–268.79 ± 3.04 μM, when compared to the reference acarbose. Amongst them, compound **68p** was found to be the most active inhibitor with an IC_50_ value of 2.56 ± 0.08 μM. On the other hand, enzyme kinetic studies describe that compound **68p** acts as a noncompetitive inhibitor with a Ki of 2.14 μM. SAR analysis divulged that the presence of electron-withdrawing groups at the 5-position of the isatin nucleus led to an increase in the inhibitory potential. It is noteworthy that most of the active analogues with different substituted benzyl groups at the N-1 position of the isatin framework exhibited excellent α-glucosidase inhibitory activity, while the introduction of an electron-donating methyl group at N-1 or C-7 positions of the isatin ring led to a decrease in potential. A docking study revealed the coumarin ring of **68p** shows an arene cation interaction with Arg-312, while the indole ring showed π-π stacking with the amino acid residue Phe-157 and formed a CH-π interaction with Phe-177. Moreover, amino acid residues Asn-347, Tyr-344, and His-279 formed H-bond interactions with **68p**, which was an important interaction for α-glucosidase inhibition [89]. In the continuation of this work, a research group in 2017 further constructed novel isatin–thiazole hybrids (**69a**–**p**, Figure 9) for their in vitro α-glucosidase inhibitory activity. The majority of the synthesized candidates showed outstanding inhibitory potential with IC_50_ values ranging between 5.36 ± 0.13 and 35.76 ± 0.31 µm compared to the reference. The most active analogue from series **69p** had a hydroxyl group at the 4-position of the phenyl ring, and N1-positions of 5-methylisatin substituted with 2-fluorobenzyl exhibited an IC_50_ value of 5.36 ± 0.13 µm. Molecular docking studies of **69p** indicated its potency by different interactions. The Indolin-2-one ring of **69p** established a CH-π interaction with Phe-157, whereas the 4-hydroxylphenyl group displayed a CH-π interaction with Phe-158 and Tyr-71 as well as forming arene anion interactions with Asp-68 and Asp-349. In addition, arene–anion interactions were observed between Arg-439, Arg-443, and **69p**, whereas compound **69p** established H-bond interactions with Thr-215, Asp-68, and Glu-276, which were important interactions for α-glucosidase inhibition [90]. In another study endeavoring to find a potent α-glucosidase inhibitor, a group in 2018 also designed chromone–isatin hybrids (**70a**–**p**, Figure 9), which were found to display excellent a-glucosidase inhibitory potential with IC_50_ values in the range of 3.18 ± 0.12–16.59 ± 0.17 μM. Analogue **70j** bearing a 4-bromobenzyl substitution at the N-1 position of isatin and hydroxyl at the 7-position of the chromone ring exhibited the highest potency with IC_50_ 3.18 ± 0.12 μM. The obtained results suggested that the presence of substitution on the chromone ring is essential for the inhibitory potential. In addition, the N1-position of isatin is substituted by various alkyls, resulting in a slight increase or decrease in activity [91].

In 2019, Altowyan et al. [92] developed spiroindolone analogues (**71a**–**r**) for dual inhibitory potential on α-Amylase and α-Glucosidase (Figure 9). During evaluation studies, it was found that compound **71r,** bearing amino groups on the aryl ring, was found to be the most active and showed excellent α-amylase (IC_50_ 22.61 + 0.54 µM) and α-glucosidase (IC_50_ 14.05 + 1.03 µM) inhibitory potential with selectivity indexes of 0.62 and 1.60, respectively, when compared to the standard acarbose. The docking study revealed that oxoindole oxygen of compound **71r** forms a hydrogen bond interaction with TRP 193 A, whereas the oxygen of the benzofuran ring formed a hydrogen bond with the residue ASN 191 A [92]. Structural fragments essential for anti-diabetic potential are revealed in Table 1.

### 2.8. Isatin as Anti-HIV Agent

**Figure 10:**
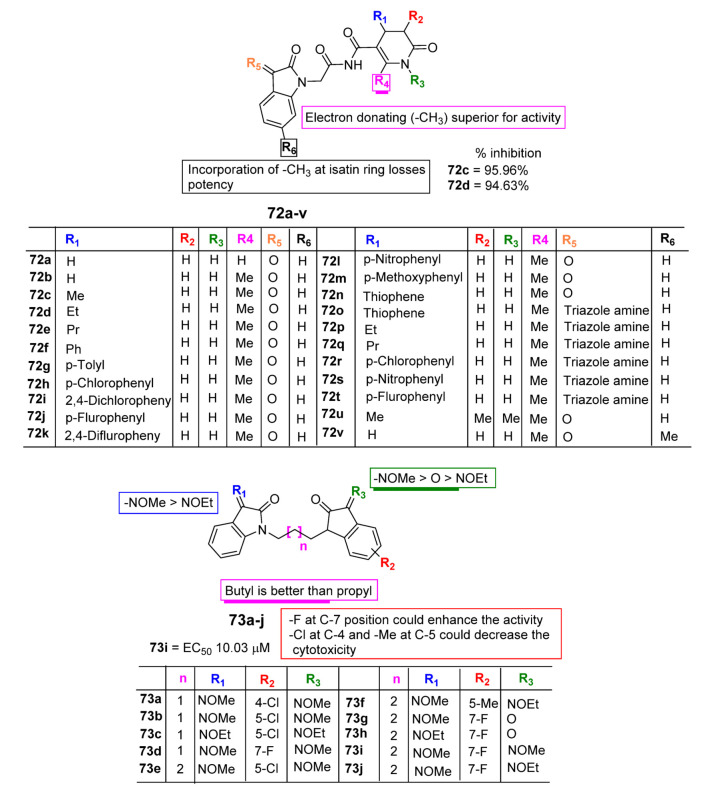
SAR outlines for anti-HIV isatin derivatives.

To develop potential anti-HIV building blocks, Devale et al. [93] in 2016 prepared novel dhydro-pyrimidinone-Isatin analogues (**72a**–**v**) as non-nucleoside HIV-1 Reverse Transcriptase inhibitors. The in vitro evaluation study proposed that all furnished analogues displayed a percent inhibition in the range of 40 to 96%. Among them, analogues **72c** and **72d** exhibited the highest percent inhibition of 95.96% and 94.63%, respectively, compared to the reference rilpivirine (94%). SAR analysis indicated the unsubstituted isatin and dihydropyrimidinone **72a** possessed the lowest percent inhibition 59% in an RT assay, whereas incorporation of the methyl group on the R-4 position of dihydropyrimidinones **72b** slightly enhances the potential, while a methyl group substituted at the phenyl ring of the isatin moiety **72v** resulted in a loss of potency. A loss of potential was seen when a methyl group was incorporated at R-2 and R-3 positions like the corresponding **72u**. Thus, the R-4 position of dihydropyrimidinone substituted with an electron-donating methyl group shows promising potency for further design and development (Figure 10). Zhang et al. [94] in 2019 investigated the anti-HIV activity of some new heteronuclear bis-isatin scaffolds tethered through propyl and butyl (**73a**–**j**, Figure 10). The anti-HIV evaluations were performed on HIV-1IIIB (MT-4) cells. All tested heteronuclear dimers were found to be active against HIV-1IIIB with EC_50_ values ranging between 10.03 and 95.47 μM with accepted cytotoxicity against MT-4 cells. In contrast, compound **73i** exhibited promising activity with EC_50_ 10.03 μM against HIV-1IIIB. The SAR analysis revealed that the length of the linker group greatly influences the anti-HIV potential, and the relative contribution is butyl > propyl. The substituted C-3, C-4, and C-5 or C-7 positions of the isatin motif greatly influences the potential. -NOMe at the C-3 position of isatin was favorable towards activity whereas -NOEt was unfavorable compared to ketone hybrids. The incorporation of fluoro at C-7 or chloro at the C-5 position of the isatin motif enhances the potential, while the substitution of chloro at the C-4 position or the replacement of chloro to methyl shows a loss of potential. The anti-HIV potential and possible substitution on the isatin nucleus are displayed in Table 1.

## 3. SAR Study for Promising Actions

**Figure 11:**
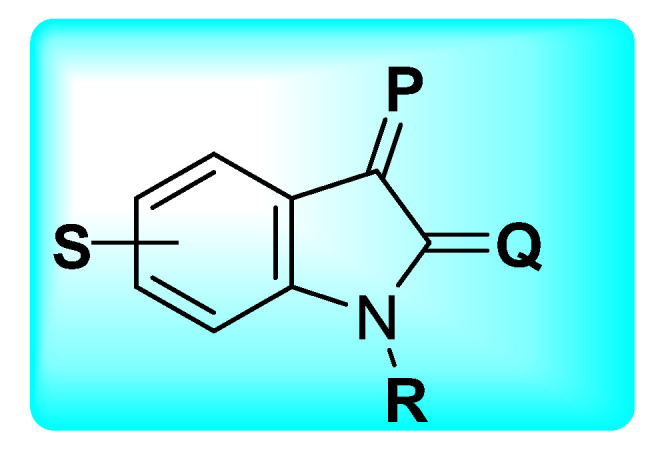
Isatin motif with its substituting positions.

The SAR features derived from various reported studies for promising activity against various infectious and non-infectious diseases are summarized in Figure 11 and Table 1. Derivatization at various positions is generally favorable for a diverse activity profile as well as to regulate pharmacokinetic properties.

## 4. Clinical Developments

The process of transferring the drug successfully from the preclinical to clinical stage is long and very few candidates are approved. Some the most promising isatin derivatives, namely Sunitinib, Toceranib, Nintedanib, Orantinib, Semaxinib, and Hesperadin, have been approved for anticancer applications, while efforts are underway to reduce the undesirable side effects associated with the approved drugs. The major outcomes are summarized in Table 2.

## 5. Conclusions

Isatin is extensively used in the synthesis of diverse organic molecules because it can be easily synthesized by different methods in good yields, through the condensation of various substituted aliphatic/aromatic amines in a hot medium. Isatin is present in many of the heterocyclic and non-heterocyclic compounds and complexes that are of wide significance because of their diverse organic and clinical applications. This study is an attempt to discuss the rationalized information of isatin derivatives exhibiting multitherapeutic efficiency such as anticancer, anti-TB, antimicrobial, anticonvulsant, antioxidants, anti-inflammatory, antidiabetic, and anti-HIV effects.

Some of the findings regarding molecular targets for isatin analogues could be concluded based on the cited literature. The anticancer properties of isatin have been evaluated in renal, sarcoma, pancreatic, kidney, melanoma, liver, colon, leukemia, lung, and breast cancer. The targets analyzed for diverse isatin analogues include CDK2, Bax, Bcl2, EGFR, VEGFR-2, and caspase 3/7. Clinically evaluated isatin analogues, namely, sunitinib, toceranib, nintedanib, orantinib, and semaxinib, have been exhibited to target VEGF, VEGFR, PDGF, PDGFR, KIT, SCF, CD135, FLT3L, and the FMS-like tyrosine kinase 3 ligand. Some of the reported structural features to improve anticancer activity include halogenation of the aromatic ring, C-2 dimers, and C-3 reactive carbonyl groups for kinase inhibition, and the aliphatic/aromatic chain at the N-position favors microtubule destabilization. Isatin analogues could exhibit anti-inflammatory action by targeting COX-2 and altering the TNF-a-induced expression of ICAM-1, while for antidiabetic action, it acts by inhibiting alpha-amylase and alpha-glucosidase. Anticonvulsant isatin analogues have been evaluated for establishing neurotoxicity profile.

In order to provide an attractive scaffold for novel, safe, effective, and economic therapeutics approaches, hybridization with other pharmacophores, prodrugs, and metal complexes can be suggested. Isatin, a versatile nucleus in the field of medicinal chemistry, must serve as a future therapeutic lead for developing various biological agents by structural modifications or alterations on isatin scaffolds, which can be utilized to develop potentially active agents in future investigations.

## Figures and Tables

**Table 1:** Summary of SAR analysis for potential activity.

Activities	P	Q	R	S
Anti-Cancer	Carbonyl species to form hydrazone, imines, or hydrazide resulting in potent CDK and Kinase inhibition. Addition of Imidazole and pyrrole leads to potent inhibition of receptor tyrosine kinase (RTK).	Hybrids with styrenes with EDG antagonize tyrosine kinase. Dimers enhance inhibition of tyrosine kinase CDK1 and 2.	Aromatic or aliphatic substitution shows courtesy towards microtubule destabilization. Para or meta substitution good for potency compared to ortho.	Halogen species favor antitumor potential
Anti-TB	Monosubstituted bis-isatin hybrids were more active than the bis-substituted and unsubstituted hybrids (NNHCSNH_2_ > O > NOMe > NOEt > NOH).	Carbonyl function is important for potency. Unsubstituted carbonyl function Improve the potential	C-1 positions of isatin moiety are deemed as the most favorable sites for hybridization. Incorporation alkyl chain linker, triazole linker displayed potent anti-TB action.	Electron withdrawing substitution at C-5 position of isatin resulted in excellent anti-TB potency.
Anti-Microbial	Schiff bases, thio-semicarbazones, semicarbazones, substituted oximes, hydrazones, etc., good for promising antimicrobial action.	Unsubstituted Q position favors noncovalent inactions at binding site.	Unsubstituted or alkyl chain linker, triazole linker resulted in good activity.	Aryl ring substituted with EWGs exhibited excellent antimicrobial action, occasionally EDGs are also good.
Anti-Convulsant	Schiff bases, thiosemicarbazones, semicarbazones with substituted aryl ring good for anticonvulsant potential.	Free to bind with receptor cavity.	Substitution at R eliminated the anticonvulsant action.	Substitution of small EWGs exhibited potential activity.
Anti-Oxidant	Imine, hydrazone, spiro, oxime oxindole substitution favors the antioxidant action.	Hydrogen bonding domain.	N-alkylation, aryl, acyl unfavorable for antioxidant potential.	EWGs such as halogens, -NO2 atC-5 increase significant enhancement in activity.
Anti-Inflammatory	Incorporation of EDGs substituted phenylhydrazone at C-3 position displayed added anti-inflammatory activity.	Hydrogen bonding domain.	Smaller N-substitution favors the anti-inflammatory potential.	EWGs at C-5 position of isatin motif favorable for anti-inflammatory action compared to EDGs.
Anti-Diabetic	Schiff bases, semicarbazones, and hydrazone link EDGs-substituted aryl, phenyl chromine ring promising for inhibitory action.	Hydrogen bonding domain.	N-substitution favors the anti-diabetic potential.	EWGs favor the inhibitory potential when compared to EDGs.
Anti-HIV	Schiff bases, semicarbazones, and hydrazone oximes could boost the potency.	No substitution is preferred.	N-substitution such as alkyl linker, triazole could improve anti-HIV potential.	EWGs at C-5 position could enhance anti-HIV profile.

**Table 2:** Summary of clinical trials and approval for isatin derivatives according to ClinicalTrials.gov database [95].

Name of Drug	Year	FDA Approved Clinical Indications
Sunitinib	2006	Gastrointestinal stromal tumors and advanced renal cell carcinoma
	2011	Pancreatic cancer
	2017	Adjuvant agent for recurrent renal carcinoma
	2019	Phase 2 in metastatic pancreatic neuroendocrine tumor
	2020	Metastatic renal cell carcinoma
Toceranib	2009	Canine mast cell tumor
Nintedanib	2018	Phase 3 completed for refractory metastatic colorectal cancer
	2018	Phase 3 completed for combination with Paclitaxel and Carboplatin for use in ovarian cancer (first line therapy)
	2018	Phase 3 completed for combination with Docetaxel for use in non-small cell lung cancer
	2019	Phase 1 completed for combination with Letrozole for breast cancer in postmenopausal women
	2019	Phase 2 completed for recurrent or metastatic breast cancer
	2019	Phase 2 terminated for metastatic HER2-negative inflammatory breast cancer
	2019	Phase 2 completed for advanced ovarian cancer
Orantinib	2011	Phase1/2 completed for use in advanced hepatocellular carcinoma
	2017	Phase 3 in hepatocellular carcinoma
Semaxinib	2003	Phase 2 completed for use in persistent and recurrent cervical cancer
	2004	Phase 3 completed for use as combination with 5-Fluorouracil, Leucovorin, and Irinotecan in metastatic colorectal cancer
	2009	Phase 2 completed for use in advanced/recurrent head and neck cancer

## Data Availability

Data sharing is not applicable in the article.

## Abbreviations

Bcl-2: B-cell Lymphoma 2; Caco-2: Human Colorectal Adenocarcinoma Cells; CCK-8: Cell Counting Kit-8; CD135: Cluster of differentiation antigen 135; CDK2: Cyclin-dependent Kinase 2; COX-1: Cyclooxygenase 1; COX-2: Cyclooxygenase 2; CPFX: *Ciprofloxacin;* CyA: Cynoscion Arenarius-1; EAC: Ehrlich-Lettre Ascites Carcinoma; EGFR: Epidermal Growth Factor Receptor; FLT3L: FMS-like tyrosine kinase 3 ligand; FMS: Fibromyalgia syndrome; GES-1: Human Gastric Epithelial Cell; GSH-Px: Glutathione peroxidase; HepG2: Liver Hepatocellular Carcinoma Cell; HIV: Human Immunodeficiency Virus; ICAM-1: Intercellular Adhesion Molecule; INH: Isoniazide; LNCaP: Lymph Node Carcinoma of the Prostate; MDR-TB: Multidrug-resistant TB; MTB: *Mycobacterium tuberculosis*; PDGF: Platelet-derived growth factor; PDGFR: Platelet-derived growth factor receptor; RIF: Rifamycin; SOD: Superoxide dismutase; TB: Tuberculosis; VEGF: Vascular Endothelial Growth Factor; VEGFR-2: Vascular Endothelial Growth Factor-2.
